# Fast perceptual learning induces location-specific facilitation and suppression at early stages of visual cortical processing

**DOI:** 10.3389/fnhum.2024.1473644

**Published:** 2025-01-17

**Authors:** Yajie Wang, Zhe Qu, You Wang, Mingze Sun, Mengting Mao, Yulong Ding

**Affiliations:** ^1^School of Psychology, Center for Studies of Psychological Application, Guangdong Key Laboratory of Mental Health and Cognitive Science, Ministry of Education Key Laboratory of Brain, Cognition and Education Sciences, South China Normal University, Guangzhou, China; ^2^Department of Psychology, Sun Yat-Sen University, Guangzhou, China; ^3^Department of Psychology, School of Public Health, Southern Medical University, Guangzhou, China

**Keywords:** fast perceptual learning, location specificity, spatial attention, target facilitation and distractor suppression, P1, ERP

## Abstract

Tens of minutes of training can significantly improve visual discriminability of human adults, and this fast perceptual learning (PL) effect is usually specific to the trained location, with little transfer to untrained locations. Although location specificity is generally considered as a hallmark of visual PL, it remains unclear whether it involves both facilitation of trained locations and suppression of untrained locations. Here we developed a novel experimental design to investigate the cognitive neural mechanism of location specificity of fast PL. Specifically, we manipulated attentional settings and recorded event-related potentials (ERPs) in both the training and tests. To get reliable location-specific PL effects on early ERPs, we adopted a new approach involving analysis of contralateral-minus-ipsilateral P1 (P1c-i). ERP results showed that tens of minutes of training not only increased the late P1c-i (~100–120 ms) evoked by targets at the trained location, but also decreased the early P1c-i (~75–95 ms) evoked by distractors at the untrained location, both of which were location specific. Moreover, comparison between the pretest and posttest revealed that the suppression effect of early P1c-i preserved even when the untrained location became target location, whereas the facilitation effect of late P1c-i appeared only when the trained location remained actively attended. These findings provide the first evidence that fast PL induces both location-specific facilitation and location-specific suppression at early stages of visual cortical processing. We speculate that while the facilitation effect indicates more efficient allocation of voluntary attention to the trained location induced by fast PL, the suppression effect may reflect learning-associated involuntary suppression of visual processing at the untrained location. Several confounding factors with regard to the early ERP effects of PL are discussed, and some important issues worth further investigation are proposed.

## 1 Introduction

The ability to discriminate small changes in sensory attributes improves remarkably with practice, a process referred to as perceptual learning (PL). PL has been found to be specific to simple stimulus attributes (e.g., location and orientation) in various visual discrimination tasks (e.g., Ahissar and Hochstein, 1997; Crist et al., 1997; Fahle et al., 1995; Karni and Sagi, 1991; Poggio et al., 1992; for reviews, see Fahle, 2005; Gilbert et al., 2001; Li, 2016; Sagi, 2011). For example, training of discriminating visual stimuli presented at one location of the visual field enhanced behavioral performance significantly, but this learning effect did not transfer to another location, regardless of whether there were distractors presented at this untrained location during the training (e.g., Gutnisky et al., 2009; Karni and Sagi, 1991) or not (e.g., Crist et al., 1997; Fahle et al., 1995). So far, the location specificity of PL has been observed in various visual tasks using different types of stimuli (Ahissar and Hochstein, 1997; Jia et al., 2020; Schoups et al., 1995; Shiu and Pashler, 1992), in both tens of minutes of training (fast learning, e.g., Fahle et al., 1995; Shiu and Pashler, 1992) and extensive training over several days (slow learning, e.g., Harris et al., 2012; Karni and Sagi, 1991; Yotsumoto et al., 2008). The striking specificity of PL to stimulus location leads to a speculation that learning-induced changes take place in relatively early stages of visual cortical areas where neural activities are highly selective for stimulus location (for reviews see Ahissar and Hochstein, 2004; Gilbert et al., 2001; Sagi, 2011; Watanabe and Sasaki, 2015).

Though the location specificity of PL is generally considered as a hallmark of visual PL (e.g., Lu and Dosher, 2022; Lu et al., 2011; Maniglia and Seitz, 2018), the underlying mechanism remains unclear. An important question is whether the location specificity of PL reflects facilitation of processing at the trained location only, or involves suppression of processing at the untrained locations as well. It is notoriously difficult to figure out this question using behavioral measurements, because both learning-induced facilitation and suppression mechanisms would lead to the same behavioral outcomes. For example, in a typical PL study of location specificity (e.g., Ahissar and Hochstein, 1997; Crist et al., 1997; Fahle et al., 1995; Gutnisky et al., 2009; Karni and Sagi, 1991), behavioral performance at the trained location improved significantly, while that at untrained locations did not change or only improved a little after training. Traditionally, such results are attributed to facilitative processing specific to the trained location. However, an alternative hypothesis could not be excluded: although visual discriminability is improved by learning, it could not be reflected at untrained locations due to some sort of learned suppression to those locations. According to literatures, PL may be a complex process (Ding et al., 2003; Fahle, 2005; Ahissar and Hochstein, 2004) involving not only stimulus-specific mechanisms but also general learning mechanisms (such as learning of task rule or task familiarity which are essential for discriminability improvement in a specific visual task; Qu et al., 2014; Zhang et al., 2010). If there is learning-induced suppression to the untrained stimulus attribute (e.g., location or orientation), it would inhibit the expression of the general learning mechanisms, leading to little transfer of learning effects on behavioral performance in the untrained stimulus condition. Behavioral and ERP studies have shown evidence supporting that besides stimulus specificity, generalization is also common in PL (Liu and Weinshall, 2000; Qu et al., 2014). For example, in our previous fast PL studies, while behavioral results showed typical orientation-specific learning effects, ERPs revealed not only specific effects but also generalized effects (as reflected by different ERP components; Ding et al., 2003; Song et al., 2007). Through double training or training-plus-exposure paradigms (e.g., Xiao et al., 2008; Xiong et al., 2016; Zhang et al., 2010; Zhang and Yang, 2014), researchers proposed that learning specificity may result from under-activations of untrained visual neurons responding to untrained locations, and training visible stimuli or exposing invisible stimuli at untrained locations may activate the untrained visual neurons, leading to transfer of learning at untrained locations (Xiong et al., 2016). However, evidence is lacking with regard to whether the untrained locations are merely not activated (while the trained locations are activated) or even suppressed during the training. Till now, it remains unclear, whether location-specificity of PL involves both facilitation of processing at the trained location and suppression of processing at the untrained location.

Here we propose that it’s possible to dissociate these two mechanisms through neural measures. Specifically, ERP measurement with its high temporal resolution and reasonable spatial resolution may provide key insights into the underlying mechanisms driving behavior (e.g., Bao et al., 2010; Ding et al., 2003; Fahle and Skrandies, 1994; Hu et al., 2019; Qu et al., 2010; Qu et al., 2014; Qu et al., 2017; Xi et al., 2020; Zhang et al., 2015). One can record the ERPs evoked by targets and distractors during the training, respectively, and to examine whether they are modulated by learning: if the early ERP activity evoked by targets (at trained location) increases and that evoked by distractors (at untrained location) decreases significantly, it will support that both facilitation of target (or trained location) and suppression of distractor (or untrained location) are induced by PL.

If both facilitation of the trained location and suppression of the untrained location are induced by PL, another important question arises: whether they involve similar cognitive neural processes. Specifically, does the learning effect reflect changes of a voluntary attentional deployment, or an involuntary visual processing? Manipulating attentional settings when measuring the learning effects may help to dissociate these two mechanisms. If a learning effect (facilitation or suppression) reflects changes of voluntary attentional deployment, it will hold when the attentional setting in measurements is similar to the training phase and will disappear when the attentional setting is altered. By contrast, if a learning effect reflects an automatic/involuntary process, it will survive across different attentional settings in measurements. Using this method, we recently revealed that shape specificity of PL (i.e., PL effects specific to the trained non-salient shapes; Hu et al., 2019; Qu et al., 2017) involves both mechanisms; that is, learning induced an initial involuntary attentional capture followed by a later voluntary attentional deployment. To our knowledge, however, such a method has not been systematically applied in investigating the location specificity of PL. So far, only a few studies have reported fast learning effects on early visual ERPs. For example, our previous study (Wang et al., 2010) found that the amplitude of the P1, an early visual ERP component appearing around 100 ms at lateral occipital sites, increased with tens of minutes of training in a difficult visual task. It remains unclear, however, whether such fast PL effects on early ERPs were specific to the trained location and whether these effects reflect changes in voluntary attentional deployment or automatic/involuntary processes.

Combining behavioral and ERP measurements, here we aim to investigate the cognitive neural mechanism of location specificity of fast visual PL. In Experiment 1, we investigated whether fast visual PL induces two kinds of location-specific effects at early stages of visual cortical processing, including both facilitation of trained location (i.e., the target or attended location during training) and suppression of untrained location (i.e., the distractor or unattended location during training). In Experiment 2, we further examined whether fast PL-induced facilitation of trained location and suppression of untrained location (both found in Experiment 1) reflect changes in voluntary attentional deployment or involuntary/automatic processing. To get reliable location-specific PL effects on early ERPs and track these changes across training at both the trained and untrained locations, we employed a new approach involving analysis of contralateral-minus-ipsilateral P1 responses.

## 2 Experiment 1

To examine whether fast PL induces both facilitation of the trained location and suppression of the untrained location, we developed an ERP experimental design that combined a sustained spatial attention paradigm (Hillyard and Anllo-Vento, 1998; Mangun and Hillyard, 1987) and fast PL paradigm of Vernier task (Fahle et al., 1995; Poggio et al., 1992). Throughout the tens of minutes of training, subjects were required to discriminate the direction of Vernier offset in one visual-field location (i.e., the trained location) and ignore Vernier stimuli in the other location (i.e., the untrained location). In addition to behavioral performance at the trained location, ERPs elicited by Vernier stimuli as targets at the trained location and as distractors at the untrained location were recorded, which allowed us to observe learning-induced changes of early visual processing at these two locations. In particular, we want to examine whether early ERPs at the trained location increased with training while those at the untrained location decreased with training. Such a design could also allow us to reveal whether and how spatial attentional effect on early ERPs (i.e., ERP difference between the trained and untrained locations) is modulated by fast learning.

### 2.1 Methods

#### 2.1.1 Subjects and apparatus

We looked to the existing literature that has examined the early ERP learning effects (e.g., Bao et al., 2010; Wang et al., 2010; Wang et al., 2016) or the sustained spatial attention effects in early ERPs (e.g., Berchicci et al., 2019; Clark and Hillyard, 1996) for guidance, which helps to estimate the necessary sample size for the present experiment. Sixteen right-handed subjects (9 female; ages 18–22 years) with normal or corrected-to-normal vision participated in Experiment 1. All were compensated for their participation, either with payment or credit hours fulfilling a course requirement. The study was conducted according to the Code of Ethics of the World Medical Association (Declaration of Helsinki) and informed written consent was obtained from each subject before the beginning of the experiment.

Experiment 1 was conducted in a dimly lit and sound-attenuated booth. All stimuli were generated and scripted using a MATLAB toolbox Psychtoolbox-3 (Brainard, 1997). Visual stimuli were presented on a 17-inch CRT monitor (Dell), with a resolution of 1,024 × 768 and a refresh rate of 60 Hz. Auditory stimuli were presented by a pair of loudspeakers placed at the left and right sides of the monitor. Subjects’ head position was stabilized with a chin rest at a viewing distance of 100 cm.

#### 2.1.2 Stimuli and task

**Figure 1:**
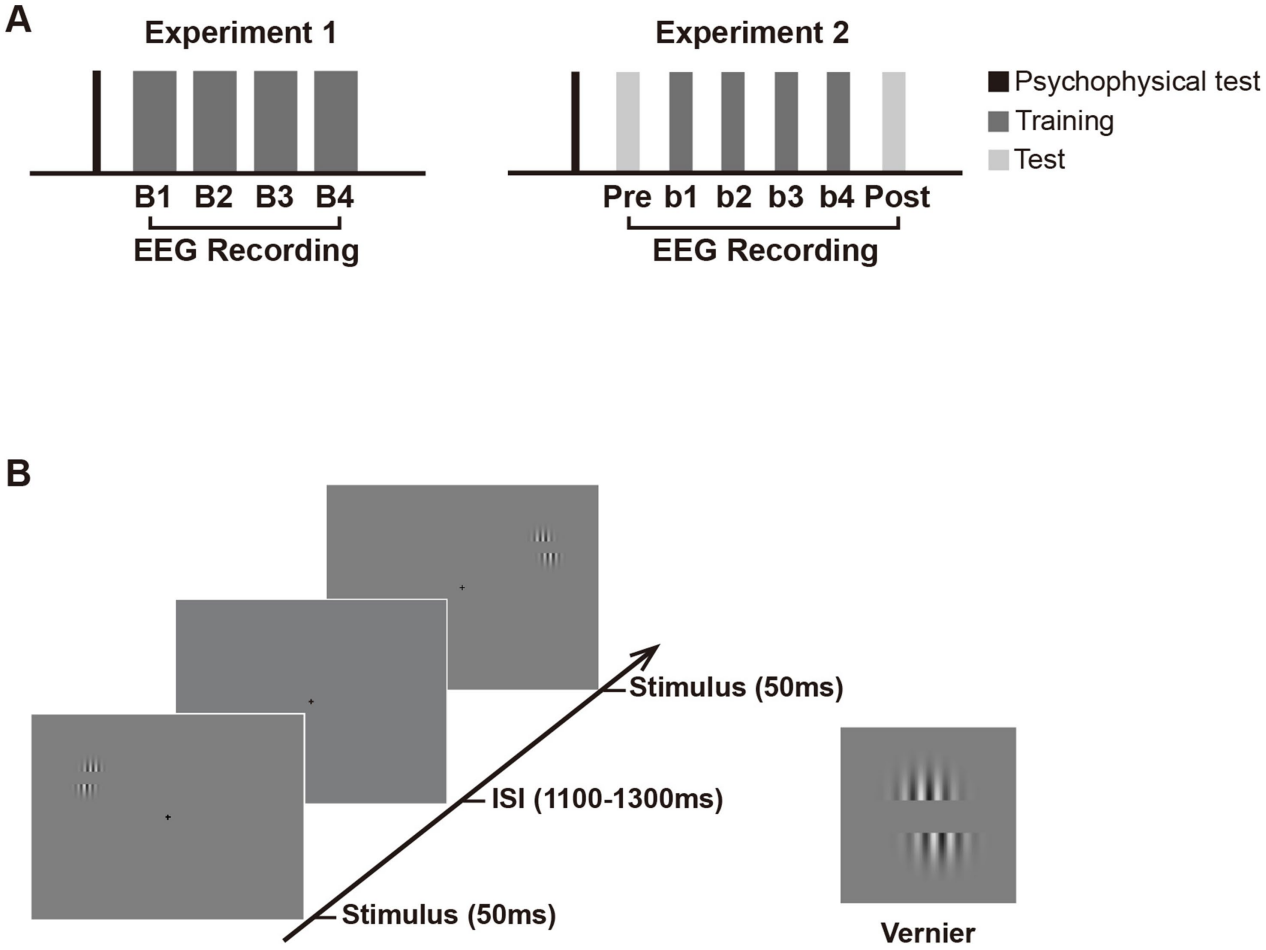
Schematic presentation of the experimental design. **(A)** Experimental procedure. Experiment 1 consisted of a psychophysical test and four training blocks (B1 to B4) with EEG recording. Experiment 2 consisted of an EEG test before (pretest) and after training (posttest), in addition to similar psychophysical test and training blocks as in Experiment 1. Note that in Experiment 2, the number of trials in each training and test block is half of that in each block in Experiment 1 to avoid fatigue effect. **(B)** Trial sequence in the training and test phase. In both experiments, Vernier stimulus was briefly flashed (50 ms) at the left or right visual fields with equal probabilities. The inter-stimulus intervals (ISIs) ranged from 1,100 to 1,300 ms. Subjects were required to judge whether the lower grating was to the left or right of the upper grating in one visual hemifield (i.e., the trained location) throughout training and ignore the other hemifield (i.e., the untrained location). They were to respond as accurately and quickly as possible once the Vernier stimulus appeared. If the position of the lower grating was offset to the left of the upper grating, participants were required to press ‘1’ on the numeric keypad; if the lower grating was offset to the right of the upper, they pressed ‘3’. Auditory feedback was given immediately after behavioral response (lower pitched sound for correct response and higher pitched sound for incorrect response). In Experiment 2, the Test and training blocks used identical stimulus displays and only differed by the instructions given to subjects. In the pretest and posttest, subjects were instructed to discriminate the Vernier offset at the untrained location and ignore that at the trained location.

In Experiment 1, subjects participated in a four-block training of a simple Vernier task (Figure 1A), in which they were required to discriminate the direction of horizontal offset in the Vernier. As shown in Figure 1B, the Vernier stimulus comprised a pair of identical sinusoidal gratings [diameter = 2.75°, contrast = 0.8, spatial frequency = 3 cpd, orientation = vertical, and a center-to-center distance = 2 *λ* (1 λ ≈ 0.34°)] on a gray background (mean luminance, 9.86 cd/m^2^). The position of the lower grating was offset to the left or right of the upper grating with equal probabilities, and the offset size was determined by the psychophysical test before training for each subject. In each “training” block, the Vernier appeared either in the upper left visual field (LVF) or the upper right (RVF) at 5° retinal eccentricity. Specifically, Vernier’s center was located 2.11° above the horizontal line of the screen and 4.53° from the vertical center line. Each Vernier was presented for 50 ms, with a 1,100–1,300 ms interval between successive stimuli (Figure 1B). To avoid repeated appearance of stimuli at the same location for more than 3 consecutive trials, the stimuli were presented at the trained or untrained location in a pseudo-random order. A small black cross in the center of a screen (0.3° × 0.3°; 0.23 cd/m^2^) was present throughout the block to help with the fixation. Subjects were required to judge whether the lower grating was to the left or right of the upper grating in one visual hemifield (i.e., the trained location) throughout training and ignored the other hemifield (i.e., the untrained location). They were instructed to respond as accurately and quickly as possible. Auditory feedback was given after behavioral responses. The trained location and the untrained location were counterbalanced across subjects; that is, half of them were required to attend the LVF throughout training, while the other half were required to attend the RVF. The two locations were completely matched for stimulus display but only differed in spatial attention settings with such a design. Therefore, any differential electrophysiological responses between these locations could be attributed to the effect of spatial attention and/or fast learning. Subjects received four training blocks, each containing 640 trials (i.e., 320 trials for the attended locations and 320 for the unattended location).

Different from the classic Hillyard sustained spatial attention paradigm (e.g., Clark and Hillyard, 1996; Di Russo et al., 2003; Martinez et al., 1999), the present design required subjects to respond to each stimulus at the attended location and used a longer inter-stimulus interval (ISI). Through such a design, stable behavioral measures of fast learning could be obtained through a large number of trials, and sufficient time were provided to subjects to prepare for the next stimulus. The present design also brought up a problem that greater slow-wave activities from preceding trials (e.g., motor-related ERPs) might overlap with the early ERPs of current trials. Although a jitter of ISI could mitigate the overlapping to certain extent, it could not completely rule out the possibility of contamination from overlapping. To solve this problem, we employed a new approach using analysis of contralateral-minus-ipsilateral ERPs to minimize these overlapping (slow-wave activities) and to get reliable PL effects (For more details, see “2.1.4 Data analysis”).

Each subject was given a psychophysical test before training to determine the individual’s Vernier offset size. This test was separately implemented in the LVF and RVF. For the LVF test, the Vernier was briefly flashed only in LVF (50 ms), followed by a period of 1,600–2,000 ms during which subjects were required to discriminate the Vernier offset. The LVF test contained four blocks of 8 trials, with decreasing Vernier offset from 6 to 3 pixels (1 pixel ≈ 0.018°); that is, the offset sizes were 6 (≈0.108°), 5 (≈0.09°), 4 (≈0.072°), and 3 (≈0.054°) pixels for these four blocks, respectively. The procedure of the psychophysical test for the RVF was identical to that in the LVF, except that Vernier stimuli were only presented in the RVF in each trial. The test order (i.e., test in the LVF first vs. test in the RVF first) was counterbalanced across subjects. For each subject, behavioral performance at each offset size was collapsed across the LVF and RVF for response accuracy calculation. Then the Vernier offset size for which subjects’ discrimination accuracy fell at 75% was regarded as the fixed stimulus parameter used in the subsequent training blocks. The whole experiment (the psychophysical test and training) lasted for about 1.8 h (the training phase lasted for about 1.6 h), including participants’ breaks between blocks.

#### 2.1.3 EEG recording

The EEG was recorded from 57 scalp sites using the 10–10 system montage. Standard 10–20 sites were FP1, FPz, FP2, F7, F3, Fz, F4, F8, T7, C3, Cz, C4, T8, P7, P3, Pz, P4, P8, O1, Oz, and O2. Additional intermediate sites were AF3, AFz, AF4, F5, F1, F2, F6, FC5, FC3, FC1, FCz, FC2, FC4, FC6, C5, C1, C2, C6, TP7, CP5, CP3, CP1, CPz, CP2, CP4, CP6, TP8, P5, P1, P2, P6, PO7, PO3, POz, PO4, and PO8. All scalp channels were recorded using a common average reference online and were then algebraically re-referenced to the average of the left and right mastoids offline. The horizontal and vertical electrooculogram (EOGs) were monitored with bipolar recordings from electrodes at the left and right outer canthi, and from those above and below the left eye. Electrode impedances were kept below 5 kΩ.

The EEG analog signals were digitized at a 512-Hz sampling rate, and a digital antialiasing filter of 0.27 × the sampling rate was applied at the time of recording. After filtering the EEG signals with a digital 40-Hz low-pass filter and then a 0.1-Hz high-pass filter, epochs were extracted that included 100 ms of pre-stimulus baseline and 600 ms of post-stimulus EEG. Trials contaminated by eye blinks, or muscle potentials exceeding ±70 μV at any electrode were excluded before averaging. Besides, trials with horizontal eye movements exceeding ±30 μV in the bipolar HEOG channel (<1% of trials) were discarded by a step-like artifact detection procedure (moving window width = 200 ms, moving step = 10 ms), so that the observed early ERP effects may not be attributed to sudden shifts in eye position (i.e., saccades) triggered by stimulus onset. After artifact rejection, about 300 trials were left for each location in each block. ERPs were then averaged according to each location within each training block to examine changes in amplitudes of the ERP components with training.

#### 2.1.4 Data analysis

Accuracies in each training block were calculated for behavioral data. To examine the learning effect on behavioral performance, the accuracies were separately subjected to one-way ANOVA with the factor being training block (B1, B2, B3, and B4).

In the ERP analyses, we are interested in the early ERP changes originating from early visual cortical areas which might underlie location-specific fast PL. In each training block, early ERPs in response to the Vernier at each location (i.e., the trained or untrained location) were measured to reveal the fast learning effects. We first examined the original ERPs and found some overlapping confounds, some of which are common in fast PL studies (as well as in some visual attention studies; see Baumgartner et al., 2018; Ding, 2018; Qu and Ding, 2018; Slotnick, 2018 for reviews and comments in the special issue “Attentional modulation of early visual areas” edited by Slotnick, 2018). One source of confounds came from slow-wave activities from preceding trials, probably including response-related ERPs and/or anticipatory waveforms like the contingent negative variation, both of which originated from high-level cortical processing (see Supplementary Figure 1). In addition, some location-nonspecific training effects from higher brain areas (e.g., midline P1 effect; see Supplementary Figure 2) might also contaminate the possible location-specific effects in early visual cortical processing. These confounds overlapped with early visual evoked potentials (e.g., the C1 and lateral P1), making it hard to get reliable or interpretable location-specific PL effects on the original ERPs. Since the scalp distributions of these confounds were unrelated to the stimulus locations (e.g., the confounding activities were distributed centrally or bilaterally, with maximum amplitudes at or near the midline sites), analyses on the contra-minus-ipsilateral ERPs may eliminate these confounds to a great extent and get reliable location-specific PL effects on early ERPs (especially those on the early P1 at contralateral sites during 75–120 ms) at the trained and untrained locations, respectively. Thus, contra-minus-ipsilateral ERP waves rather than original ERPs were analyzed and reported in the present study. With such a subtraction, the first positive deflection (75–120 ms) in the contralateral-minus-ipsilateral difference wave would reflect the early visual processing of the stimulus at the trained or untrained location, which was called as ‘P1c-i’ (contra-minus-ipsilateral P1) in the present study for convenience. Through visual inspection of grand-average ERPs, we found that learning-induced P1c-i changes appeared at two different time windows (75–95 ms and 100–120 ms) for the trained and untrained locations respectively, both involving occipital sites (Figures 3C,D), which were consistent with previous findings that the contralateral P1 contains two subcomponents (early P1 and late P1; Di Russo et al., 2003). We then performed statistical analyses to test whether this observation is reliable. Specifically, the mean amplitudes of P1c-i were measured at occipital sites (PO7/8, PO3/4, P7/8, and P5/6) in two distinct time intervals (75–95 ms and 100–120 ms). The mean amplitudes of early P1c-i (75–95 ms) and the mean amplitudes of late P1c-i (100–120 ms) were then separately analyzed in a two-way ANOVA with factors being location (Trained vs. Untrained) and training block (B1, B2, B3, and B4). Two-tailed paired *t*-tests were further used if necessary.

To characterize the training-induced changes of scalp distribution of lateralized P1 (P1c-i), spline interpolated topographical maps of scalp voltage were calculated for the difference waves between training blocks (e.g., the difference in P1c-i amplitude between B1 and B2). Topographies of lateralized activity were typically projected to both sides of the head to map contralateral-minus-ipsilateral differences in previous studies (e.g., Kiss et al., 2008; Qu et al., 2014). Because such voltage maps are mirror-symmetric, only the left side of the head is highlighted in the present study (see also Störmer et al., 2019).

### 2.2 Results

#### 2.2.1 Behavioral results

**Figure 2:**
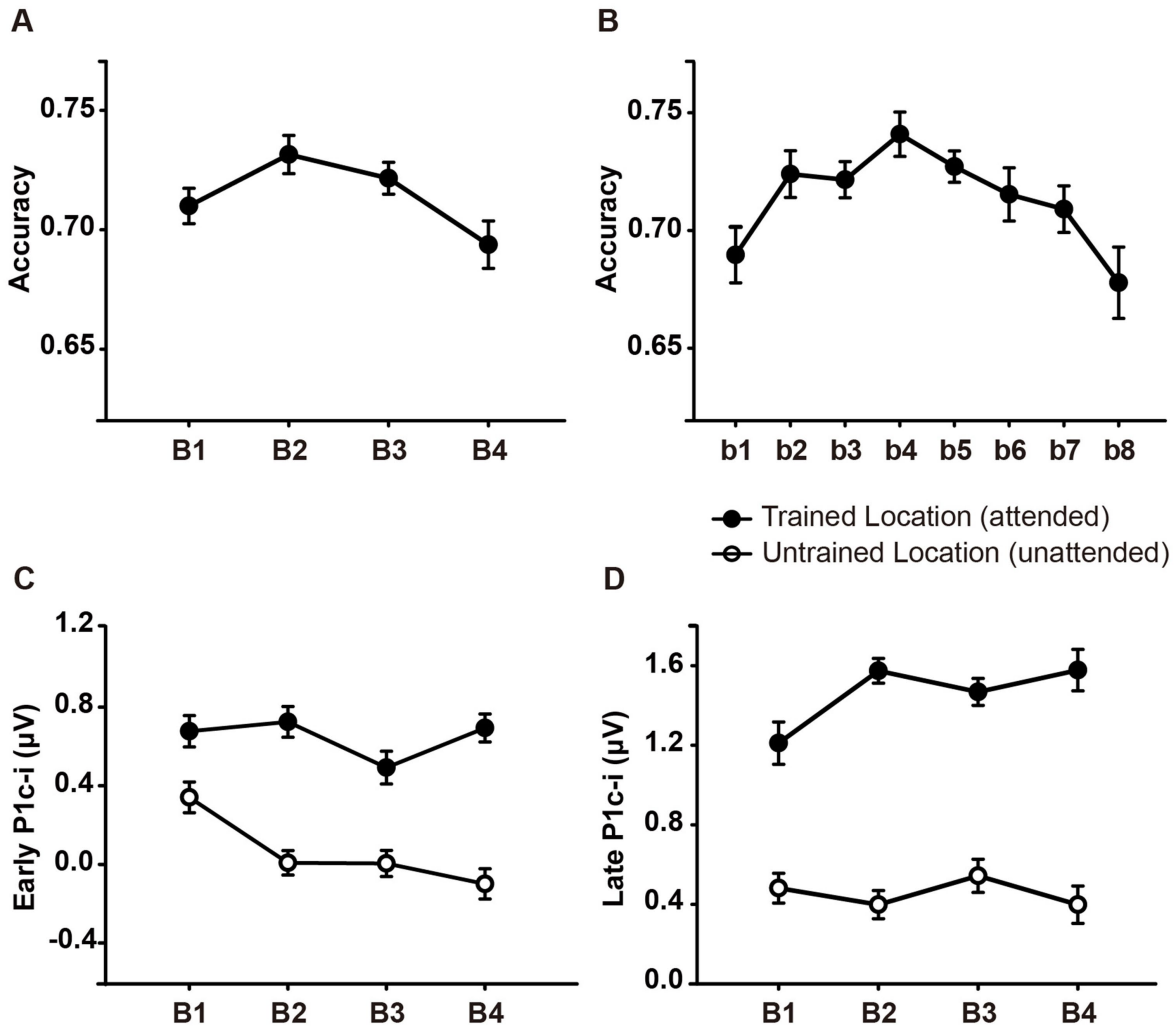
Mean accuracies and ERP amplitudes in each training block of Experiment 1. **(A)** Vernier’s discrimination accuracy at the trained location improved after a single training block of trials. **(B)** Each training block was bisected into 2 mini-blocks, resulting in 8 mini-blocks in total. There was a significant fatigue effect in the last mini-block (b8). **(C,D)** P1c-i amplitudes were measured at posterior electrodes (collapsed across PO7/8, PO3/4, P7/8, and P5/6) in two different time intervals (i.e., 75–95 ms and 100–120 ms). Training decreased early P1c-i amplitude (75–95 ms) evoked by distractors at the untrained (unfilled circles) but not that evoked by targets at the trained location (i.e., filled circles). In contrast, training increased late P1c-i amplitude (100–120 ms) at the trained but not at the untrained location. Error bars indicate within-subject standard errors (Cousineau, 2005).

As shown in Figure 2A, ~ 90 min of training induced a significant change in accuracy [main effect of training, *F* (3, 45) = 3.006, *p* = 0.040, $\eta_{p}^{2}$ = 0.167]. Further analysis revealed a trend of improvement in discrimination accuracy after a single training block of trials (B1 vs. B2 improvement, 0.022 ± 0.012, *mean* ± *SE*, *p* = 0.085, 95% CI = [−0.003, 0.046]). In the fourth training block (B4), however, the accuracy decreased significantly (B4 vs. B2, *p* = 0.043), indicating fatigue effects associated with the training. To examine the detailed time courses of learning and fatigue effects, each training block was bisected into 2 mini-blocks, resulting in 8 mini-blocks (b1 to b8) in total. As illustrated in Figure 2B, accuracy increased gradually across training and reached the maximum in the fourth mini-block (b4 vs. b1, *p* = 0.009), while fatigue effects did not reach significant until the last mini-block (b8 vs. b4, *p* = 0.016).

#### 2.2.2 ERP results

**Figure 3:**
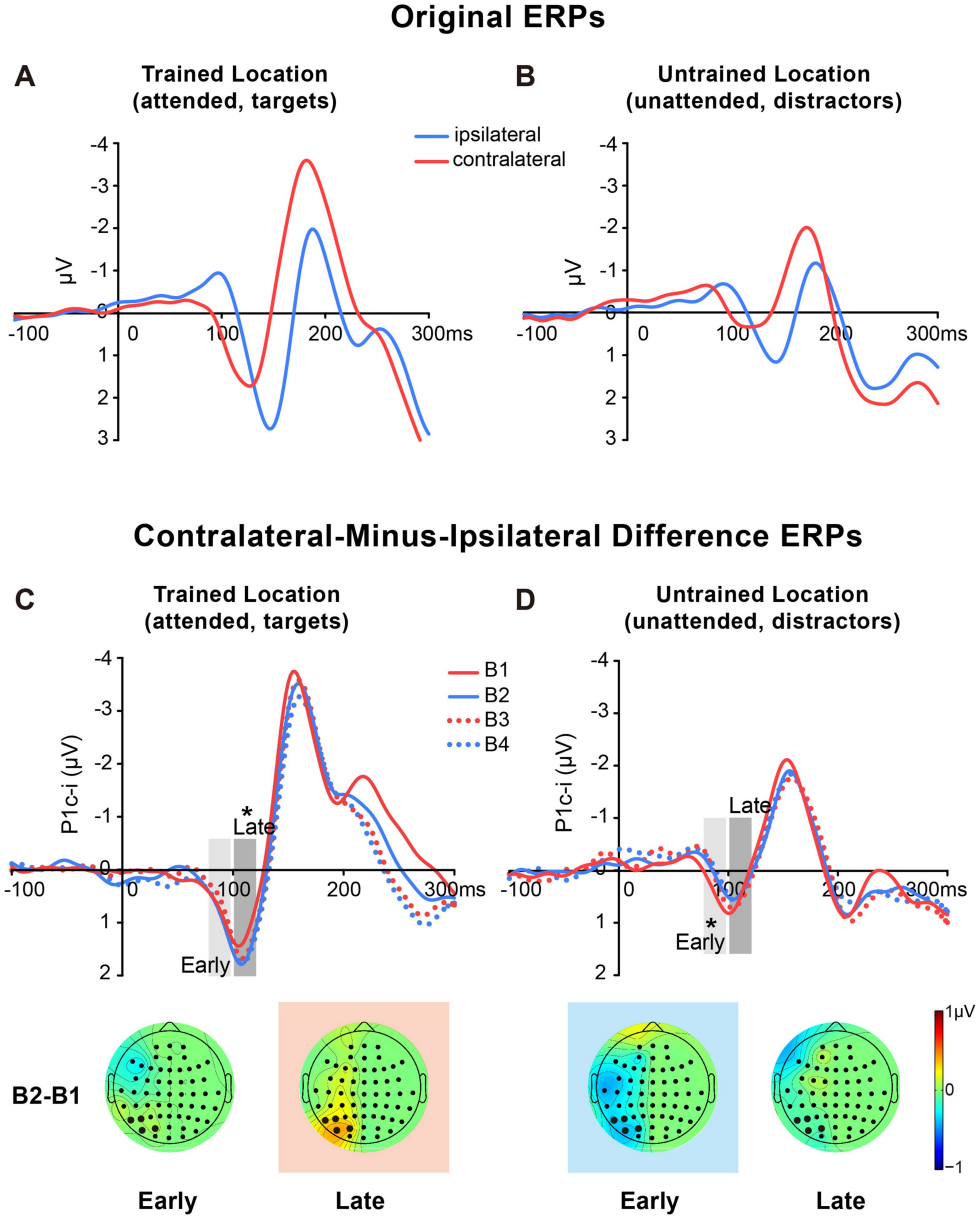
Grand average ERPs and voltage topographies in Experiment 1. **(A,B)** Original ERP waveforms (averaged across four training blocks) elicited by Vernier stimuli as targets at the trained location **(A)** and as distractors at the untrained location **(B)** at electrodes (collapsed across PO7/8, PO3/4, P7/8, and P5/6) contralateral and ipsilateral to the stimulus locations. **(C,D)** Contralateral-minus-ipsilateral difference waveform, called P1c-i, elicited by targets at the trained location **(C)** or distractors at the untrained location **(D)** in each training block (B1, B2, B3 and B4). P1c-i waves were measured in two different time intervals (i.e., 75–95 ms and 100–120 ms), shown as shaded rectangles. Topographical voltage maps of the B2 minus B1 difference amplitude averaged over the 75–95 ms and 100–120 ms time window, respectively. These voltage distributions of difference waves were projected onto the left hemisphere (for details see Data Analysis). In the maps, the electrodes highlighted in bold represent a channel group used for the analysis of learning effects, including PO7/8, PO3/4, P7/8, and P5/6. Red in the topographic plot indicates training-induced larger P1c-i, and blue indicates training-induced smaller P1c-i. * *p* < 0.05.

The original ERPs (i.e., contralateral and ipsilateral ERPs) of targets at the trained location and of distractors at the untrained location are illustrated in Figures 3A,B. Since the original ERPs contained some overlapping confounds which are common in fast PL and spatial attention studies (see Methods for details), here we measured the contra-minus-ipsilateral wave during the P1 time window, and called the difference wave as ‘P1c-i’ in the present study. Although the subtraction method might also eliminate the earliest component C1 to a large extent due to C1’s approximately midline distribution under the present stimulus condition (Di Russo et al., 2003), it could definitely exhibit differences of early P1 between contra- and ipsi-lateral sites. The P1c-i waves of targets at the trained location and those of distractors at the untrained location (Figures 3C,D) both showed a positive deflection during 75–120 ms, which is consistent with previous findings that the P1 was larger over the contralateral scalp than the ipsilateral scalp during this time window (e.g., Clark and Hillyard, 1996; Di Russo et al., 2003; Figure 1 in McDonald et al., 2022). In each training block, the amplitude of the P1c-i at each location was analyzed to track changes of brain activities during training. As shown in Figures 3C,D, the amplitude of the P1c-i at the trained location increased with training during 100–120 ms, whereas that at the untrained location decreased within 100 ms after stimulus onset.

##### 2.2.2.1 Early P1c-i

Statistical analyses of the P1c-i further confirmed the distinct learning effects at the trained and untrained locations in two time intervals. For the early P1c-i (75–95 ms), a significant interaction of location × training block was found [*F* (3, 45) = 2.986, *p* = 0.041, $\eta_{p}^{2}$ = 0.166]. Further analysis showed a training-induced significant decrease in the early P1c-i amplitudes at the untrained location [One-way ANOVA with the factor as training block, *F* (3, 45) = 5.412, *p* = 0.003, $\eta_{p}^{2}$ = 0.265; linear trend, *F* (1, 15) = 16.048, *p* = 0.001, $\eta_{p}^{2}$ = 0.517]. The decrement of early P1c-i mainly occurred between the first two blocks (B1 vs. B2 decrement, −0.333 ± 0.131 μV, *mean* ± *SE*, *p* = 0.023, 95% CI = [−0.61, −0.05]; Figures 2C,3D), and preserved in the following training blocks (B1 vs. B4, −0.440 ± 0.122 μV, *p* = 0.003, 95% CI = [−0.70, −0.18]; B2 vs. B4, −0.107 ± 0.106 μV, *p* = 0.327, 95% CI = [−0.33, 0.12]; Figure 2C). In contrast, there were no significant differences in early P1c-i amplitudes at the trained location across training blocks [*F* (3, 45) = 1.356, *p* = 0.268, $\eta_{p}^{2}$ = 0.083; Figures 2C, 3C].

##### 2.2.2.2 Late P1c-i

For the late P1c-i (100–120 ms), a significant interaction of location (Trained vs. Untrained) × training block (B1, B2, B3, and B4) was also found [*F* (3, 45) = 2.992, *p* = 0.041, $\eta_{p}^{2}$ = 0.166]. Further analysis showed that training increased the late P1c-i amplitude at the trained location significantly [One-way ANOVA with the factor as training block, *F* (3, 45) = 2.911, *p* = 0.045, $\eta_{p}^{2}$ = 0.163; linear trend, *F* (1, 15) = 3.091, *p* = 0.099, $\eta_{p}^{2}$ = 0.171]. The increase of late P1c-i mainly occurred after a single training block (B1 vs. B2 increment, 0.364 ± 0.141 μV, *mean* ± *SE*, *p* = 0.021, 95% CI = [0.06, 0.66]; Figures 2D, 3C), and almost preserved in the following training blocks (B1 vs. B4, 0.367 ± 0.187 μV, *p* = 0.068, 95% CI = [−0.03, 0.77]; B2 vs. B4, 0.004 ± 0.137 μV, *p* = 0.980, 95% CI = [−0.29, 0.30]; Figure 2D). In contrast, no significant differences in late P1c-i amplitudes were found across training blocks at the untrained location [*F* (3, 45) = 0.564, *p* = 0.642, $\eta_{p}^{2}$ = 0.036; Figures 2D, 3D].

**Figure 4:**
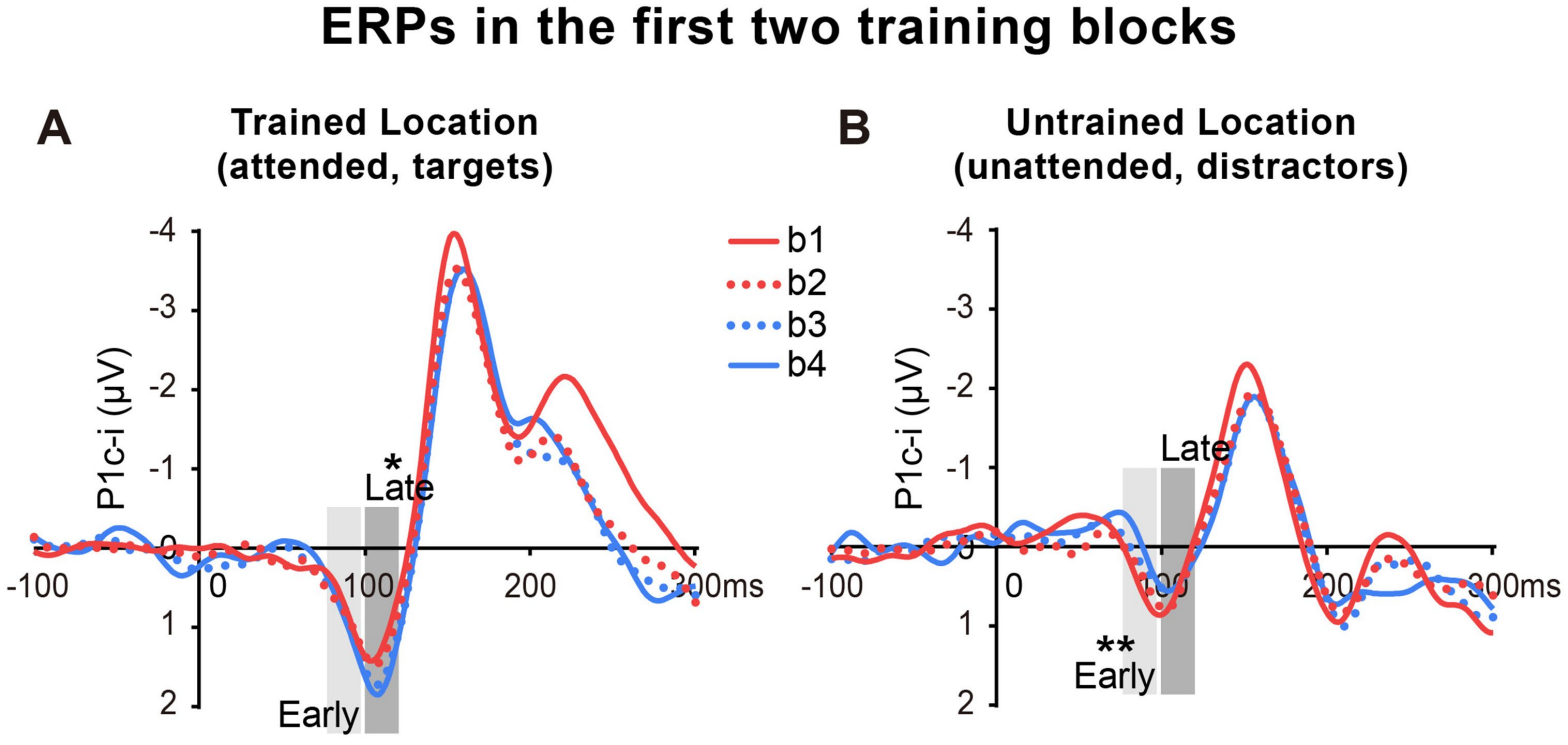
Grand average ERPs in the first two training blocks in Experiment 1. **(A,B)** The first two blocks were bisected into 4 mini-blocks (b1, b2, b3 and b4) to further examine the detailed time courses of the P1c-i learning effects, especially whether the effects appeared quickly within the first training block (i.e., after a single mini-block b1). The P1c-i elicited by targets at the trained location **(A)** or distractors at the untrained location **(B)** in each mini-block were shown. P1c-i waves were measured in two different time intervals (i.e., 75–95 ms and 100–120 ms), shown as shaded rectangles.

As reported above, both the early P1c-i effect at the untrained location and the late P1c-i effect at the trained location occurred mainly within the first two blocks and were preserved in the last block (B4). The preservation of these early ERP learning effects in B4 indicates that they are not sensitive to fatigue, which is different from the behavioral effect. Since behavioral result showed significant learning effect within the first training block (i.e., between b1 and b2), to further examine whether such quick effect also appears on the P1c-i, we divided the first two blocks into 4 mini-blocks (Figure 4). Results showed that, the early P1c-i at the untrained location decreased significantly between b1 and b4 (*p* = 0.004), but not between b1 and b2 (*p* = 0.460); similarly, the late P1c-i at the trained location increased significantly between b1 and b4 (*p* = 0.020), but not between b1 and b2 (*p* = 0.536). Taken together, the present results showed different time course of early ERP learning effects from that of behavioral effect, indicating that they involve different mechanisms. While the early and late P1c-i effects mainly reflect location-specific PL, the behavioral performance might involve not only specific PL effects but also some general effects associated with training (e.g., task-rule learning and/or fatigue).

##### 2.2.2.3 Inter-trial target/distractor effect

To examine whether the present PL effects on the P1c-i are a consequence of transient inter-trial priming (Britton and Anderson, 2020; Jiang et al., 2013; Wang and Theeuwes, 2018), we further compared the P1c-i effects between conditions when a given stimulus (target or distractor) was preceded by the same stimulus (i.e., repetition condition) or not (i.e., no-repetition condition). Results showed that training decreased the early P1c-i evoked by distractors in both distractor repetition and no-repetition conditions (*p*s < 0.02), and there was no significant difference for learning effects between these two conditions (*p* = 0.217). Similarly, for the late P1c-i at the trained location, there was no significant difference for training-induced late P1c-i increment between conditions when targets at the trained location was repeated or not (*p* = 0.888). These results suggest that inter-trial priming does not play a role in both facilitation of the trained location and suppression of the untrained location.

## 3 Experiment 2

In Experiment 1, we found that tens of minutes of training induced two location-specific effects in early ERPs: an increase of late P1c-i (~100–120 ms) evoked by targets at the trained location and a decrease of early P1c-i (~75–95 ms) evoked by distractors at the untrained location. To confirm these findings and to further examine whether these location-specific fast PL effects reflect changes in voluntary attentional deployment or involuntary/automatic visual processing, we designed Experiment 2. Subjects were given a test block both before (pretest) and after training (posttest), in addition to similar training blocks as in Experiment 1. Test and training blocks used identical stimulus displays and only differed by the instructions given to subjects: while training blocks (i.e., attend-repeat-location blocks) required subjects to discriminate the offset direction of Vernier at the trained location and ignore the untrained location, test blocks (i.e., attend-opposite-location blocks) required subjects to judge the Vernier offset at the untrained location and ignore the trained location. That is, the attentional setting of the test was different from that of the training. The trained location which was the target/attended location during the training became the distractor/unattended location in the test, whereas the untrained location which was the distractor/unattended location during the training became the target/attended location in the test.

In Experiment 2, we first expected to replicate the two PL-induced ERP effects in Experiment 1: an increase of the late P1c-i elicited by targets at the trained location and a decrease of the early P1c-i elicited by distractors at the untrained location. To a great extent, such a replication could avoid the Type I and Type II errors (Luck and Gaspelin, 2017; noted that similar to previous PL studies, early ERP effects observed in Exp. 1 show modest statistical significance, indicating that PL effects on early visual ERPs are not strong; replication would be a better approach than multiple-comparison corrected test to detect such weak signals). Then we examined whether these two effects still exist in the tests. If the target facilitation effect (indexed by the late P1c-i) could not be observed at the untrained location in the test, it will further confirm that this effect is specific to the trained location. Similarly, if the distractor suppression effect (indexed by the early P1c-i) could not be observed at the trained location in the test, it will further confirm that this effect is specific to the untrained location. In addition, since the attentional setting in the test was different from that during the training, if the late P1c-i effect at the trained location or the early P1c-i effect at the untrained location appears during the training but disappears in the test (i.e., the observation of the learning effect is dependent on the attentional setting in measurements), we may infer this learning effect reflects changes in voluntary attentional deployment. In contrast, if these early ERP learning effects appear in both the training and the test (i.e., the observation of the learning effect is independent of the attentional setting in measurements), we may infer they reflect modifications in automatic, involuntary visual processing.

### 3.1 Method

#### 3.1.1 Subjects and apparatus

We chose the same sample size as that in Experiment 1. Sixteen right-handed subjects (9 female; ages 19–29 years) with normal or corrected-to-normal vision participated in Experiment 2. The apparatus was similar to that used in Experiment 1.

#### 3.1.2 Procedure

Stimulus and procedure were similar to those described in Experiment 1 except for the following differences. All subjects were given a test block before (pretest) and after training (posttest) in Experiment 2 (Figure 1A). Test and training blocks used identical stimulus displays and only differed in the instructions given to subjects. In test blocks, subjects were required to discriminate the direction of the horizontal offset of the Vernier stimulus at the untrained location and ignore that at the trained location. The number of trials in each training block (and each test block) were reduced to half of Experiment 1 to minimize fatigue effects since two test blocks were added in Experiment 2 and significant fatigue effects were found in the last block of Experiment 1. Subjects received six blocks (i.e., four training blocks and two test blocks), each block containing 320 trials (i.e., 160 trials for the attended location and 160 trials for the unattended location). The whole experiment (the psychophysical test and training) lasted for about 1.3 h (the training phase lasted for about 1.1 h), including participants’ breaks between blocks.

#### 3.1.3 EEG recording

The EEG recordings in Experiment 2 were identical to Experiment 1 with the following exceptions. Electroencephalogram (EEG) was recorded in both training and test blocks from 58 scalp electrodes of the 10/10 system. Standard 10–20 sites in the scalp were FP1, FPz, FP2, F7, F3, Fz, F4, F8, T7, C3, Cz, C4, T8, P7, P3, Pz, P4, P8, O1, Oz, and O2. Additional intermediate sites were AF3, AFz, AF4, FC5, FC3, FC1, FCz, FC2, FC4, FC6, C5, C1, C2, C6, TP7, CP5, CP3, CP1, CPz, CP2, CP4, CP6, TP8, P5, P1, P2, P6, PO7, PO3, POz, PO4, PO8, I5, I3, Iz, I4 and I6. After artifact rejection, about 145 trials were left in each block for each location (i.e., the attended and unattended locations).

#### 3.1.4 Data analysis

In order to examine learning effects on behavioral performance, accuracies were separately subjected to one-way repeated-measure ANOVAs with the factor being training block (b1, b2, b3 and b4). In addition, a pair-wise *t*-test was used to evaluate behavioral performance at the untrained location before and after training (i.e., pre- and post-tests) for examining whether the improved performance is specific to the trained location.

ERP mean amplitudes of the P1c-i in response to Vernier stimulus at each location were measured in each block. Consistent with Experiment 1, the mean amplitudes of P1c-i were measured in two distinct time intervals (i.e., 75–95 ms and 100–120 ms) at occipital sites (PO7/8, PO3/4, P7/8, and P5/6). To replicate the two PL-induced ERP effects in Experiment 1, early and late P1c-i amplitudes at each location were then separately subjected to a one-way ANOVA with factors being training block (b1, b2, b3, and b4), respectively. Pair-wise *t*-tests between pre- and post-tests were conducted to further investigate whether these two ERP learning effects were location-specific and whether they were modified in the tests.

### 3.2 Results

#### 3.2.1 Behavioral results

**Figure 5:**
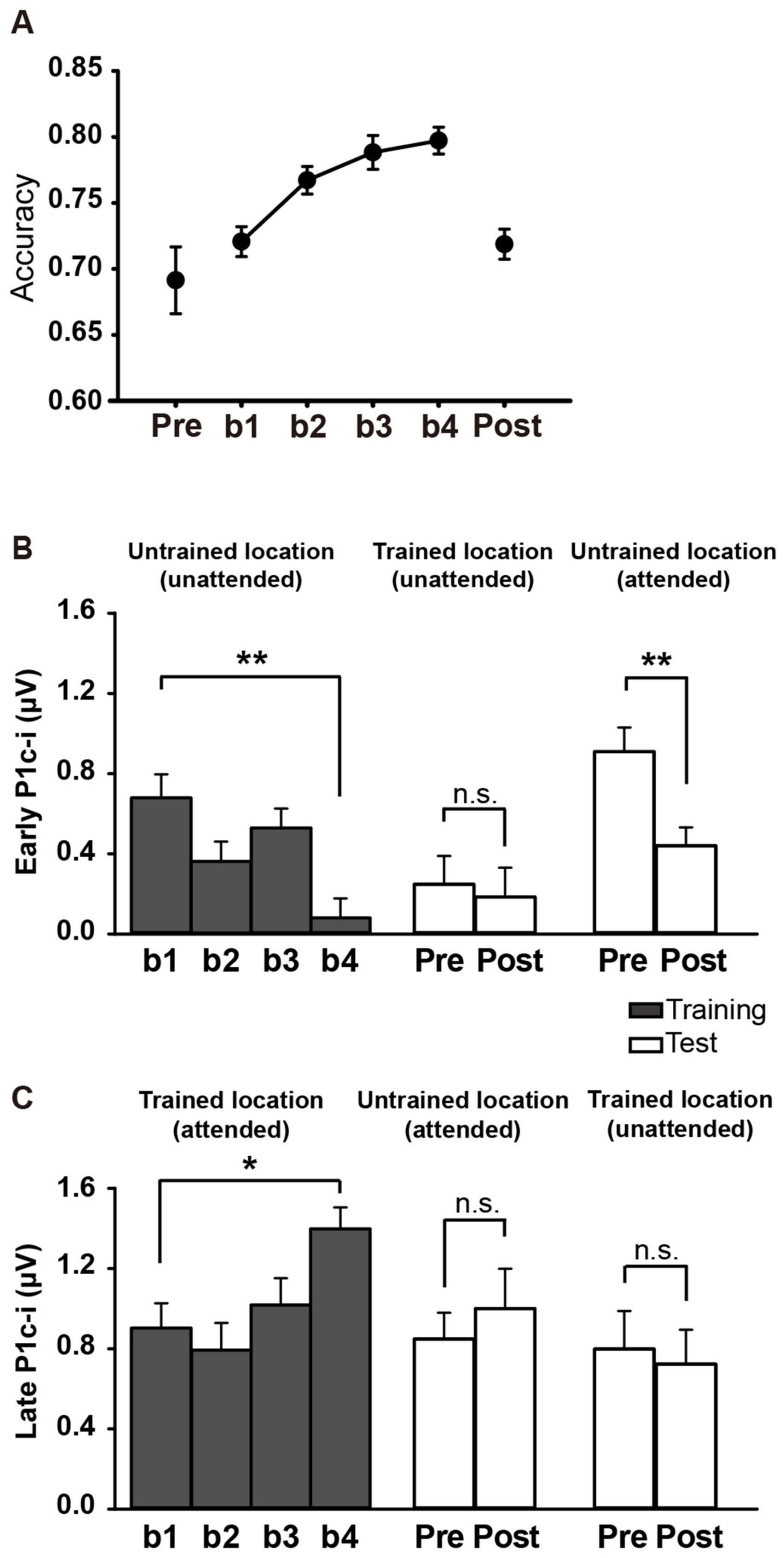
Behavioral and ERP results in Experiment 2. **(A)** Mean accuracies during training and test blocks. Behavioral performance improved significantly with training, as reflected by higher Vernier discrimination accuracy in b4, and this learning effect was specific to the trained location. **(B,C)** Mean amplitudes of the early and late P1c-i in training and test blocks. P1c-i waves were measured at electrodes (collapsed across PO7/8, PO3/4, P7/8, and P5/6) in two distinct time intervals (i.e., 75–95 ms and 100–120 ms). Training decreased the early P1c-i (75–95 ms) at the untrained location, as indexed by a smaller early P1c-i in b4 than in b1, and this effect was preserved when the untrained location became attended in the tests. The early P1c-i effect was not observed at the trained location when it was the distractor/unattended location in the tests. In contrast, the late P1c-i (100–120 ms) at the trained location increased between b1 and b4. This effect disappeared when the trained location became unattended in the tests. The late P1c-i effect was not observed at the untrained location when it was the target/attended location in the tests. * *p* < 0.05, ** *p* < 0.01, and n.s. indicates *p* > 0.5. Error bars indicate within-subject standard errors.

For all subjects, accuracies in the training and test blocks were calculated to reveal the fast PL effect and its location specificity. As shown in Figure 5A, ~60 min of training significantly improved behavioral performance [main effect of block, *F* (3, 45) = 11.258, *p* < 0.001, $\eta_{p}^{2}$ = 0.429], as reflected by the higher accuracy in b4 (b1 vs. b4 improvement, 0.077 ± 0.017, *mean* ± *SE*, *p* = 0.0004; 95% CI = [0.04, 0.11]). Consistent with previous behavioral studies showing location specificity of PL, the performance improvement disappeared at the untrained location when subjects were required to discriminate the Vernier offset at this location in the tests [pre vs. post, 0.027 ± 0.028, *t*(15) = 0.989, *p* = 0.338, *d* = 0.247; Figure 5A]. Accuracy was significantly lower in the posttest than in b4 (*p* = 0.0003), further confirming the location specificity of PL.

#### 3.2.2 ERP results

**Figure 6:**
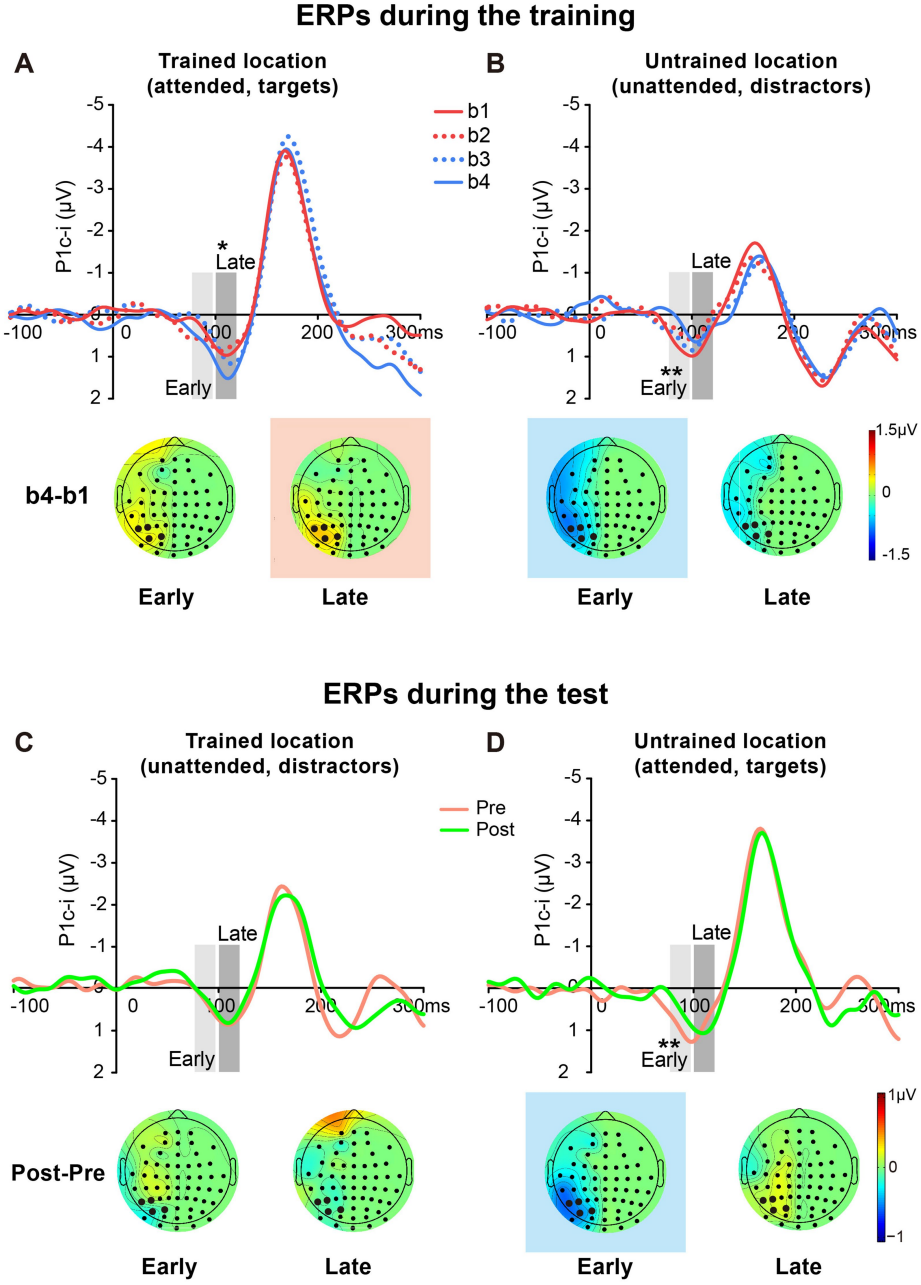
Grand average ERPs and voltage topographies in training and test blocks of Experiment 2. **(A,B)** Contralateral-minus-ipsilateral difference ERP waveforms elicited by targets at the trained location **(A)** and by distractors at the untrained location **(B)** during the training. P1c-i waves were measured in two different time intervals (i.e., 75–95 ms and 100–120 ms), shown as shaded rectangles. Topographies of these intervals were plotted based on ERP difference waves constructed by subtracting b1 from b4. These voltage distributions of difference waves were projected onto the left hemisphere. Similar to Experiment 1, training increased late P1c-i (100–120 ms) at the trained but not the untrained location, and decreased early P1c-i (75–95 ms) at the untrained but not the trained location. **(C,D)** Contralateral-minus-ipsilateral difference ERP waveform elicited by distractors at the trained location **(C)** and by targets at the untrained location **(D)** in the tests. Topographies of two intervals were plotted based on ERP difference waves between pretest and posttest. The electrodes highlighted in bold represent an electrode group (PO7/8, PO3/4, P7/8, and P5/6) used for the analysis of learning effects. Red in the topographic plot indicates training-induced larger P1c-i, and blue indicates training-induced smaller P1c-i. * *p* < 0.05, ** *p* < 0.01.

The P1c-i waves of targets at the trained location and those of distractors at the untrained location during the training were shown in Figures 6A,B, respectively. The P1c-i waves of distractors at the trained location and those of targets at the untrained location in the tests were illustrated in Figures 6C,D, respectively.

##### 3.2.2.1 Early P1c-i

Similar to Experiment 1, a significant interaction of location (Trained vs. Untrained) × training block (b1, b2, b3, and b4) was found for the early P1c-i [75–95 ms; *F* (3, 45) = 7.932, *p* = 0.002, $\eta_{p}^{2}$ = 0.346]. Again, training significantly decreased the early P1c-i amplitude at the untrained location (*F* (3, 45) = 5.673, *p* = 0.002, $\eta_{p}^{2}$ = 0.274; the decrement occurred across the four training blocks rather than after a single block: b1 vs. b4, −0.599 ± 0.171 μV, *mean* ± *SE*, *p* = 0.003, 95% CI = [−0.96, −0.24]; b1 vs. b2, −0.316 ± 0.171 μV, *p* = 0.084, 95% CI = [−0.68, 0.05]; see Figures 5B, 6B), but not at the trained location (b1 vs. b4, 0.316 ± 0.227 μV, *p* = 0.185, 95% CI = [−0.17, 0.80]; Figure 6A).

The early P1c-i decrement was not observed at the trained location in the tests [pre vs. post, −0.063 ± 0.222 μV, *t*(15) = −0.286, *p* = 0.779, *d* = −0.071; Figures 5B, 6C], reinforcing that this learning-induced suppression effect on the early P1c-i is specific to the untrained location. In addition, the early P1c-i effect observed at the untrained location during the training (Figure 6B) was preserved when the untrained location became attended in the test [pre vs. post, −0.469 ± 0.134 μV, *t*(15) = −3.499, *p* = 0.003, *d* = −0.875; Figures 5B, 6D]; that is, the early P1c-i effect appeared regardless of whether the untrained location was attended or not, indicating that this suppression effect reflects modifications in automatic/involuntary processing at the untrained location.

##### 3.2.2.2 Late P1c-i

Consistent with Experiment 1, training significantly increased the late P1c-i amplitude at the trained location (*F* (3, 45) = 4.243, *p* = 0.010, $\eta_{p}^{2}$ = 0.220; the increment occurred across the four training blocks rather than after a single block: b1 vs. b4, 0.495 ± 0.169 μV, *mean ± SE*, *p* = 0.010, 95% CI = [0.14, 0.86]; b1 vs. b2, −0.110 ± 0.204 μV, *p* = 0.597, 95% CI = [−0.55, 0.33]); Figures 5C, 6A), but not at the untrained location [*F* (3, 45) = 1.062, *p* = 0.357, $\eta_{p}^{2}$ = 0.066; Figure 6B].

The late P1c-i increment was not observed at the untrained location in the tests [pre vs. post, 0.151 ± 0.253 μV, *t*(15) = 0.599, *p* = 0.558, *d* = 0.150; Figures 5C, 6D], reinforcing that the learning-induced facilitation effect on late P1c-i is specific to the trained location. Different from the early P1c-i effect, the late P1c-i learning effect observed at the trained location during the training (Figure 6A) disappeared when the trained location became unattended in the test [pre vs. post, −0.075 ± 0.274 μV, *t*(15) = −0.275, *p* = 0.787, *d* = −0.069; Figures 5C, 6C], indicating this facilitation effect reflects changes of voluntary allocation of attention to the trained location.

##### 3.2.2.3 PL-induced changes of spatial attention effect

**Figure 7:**
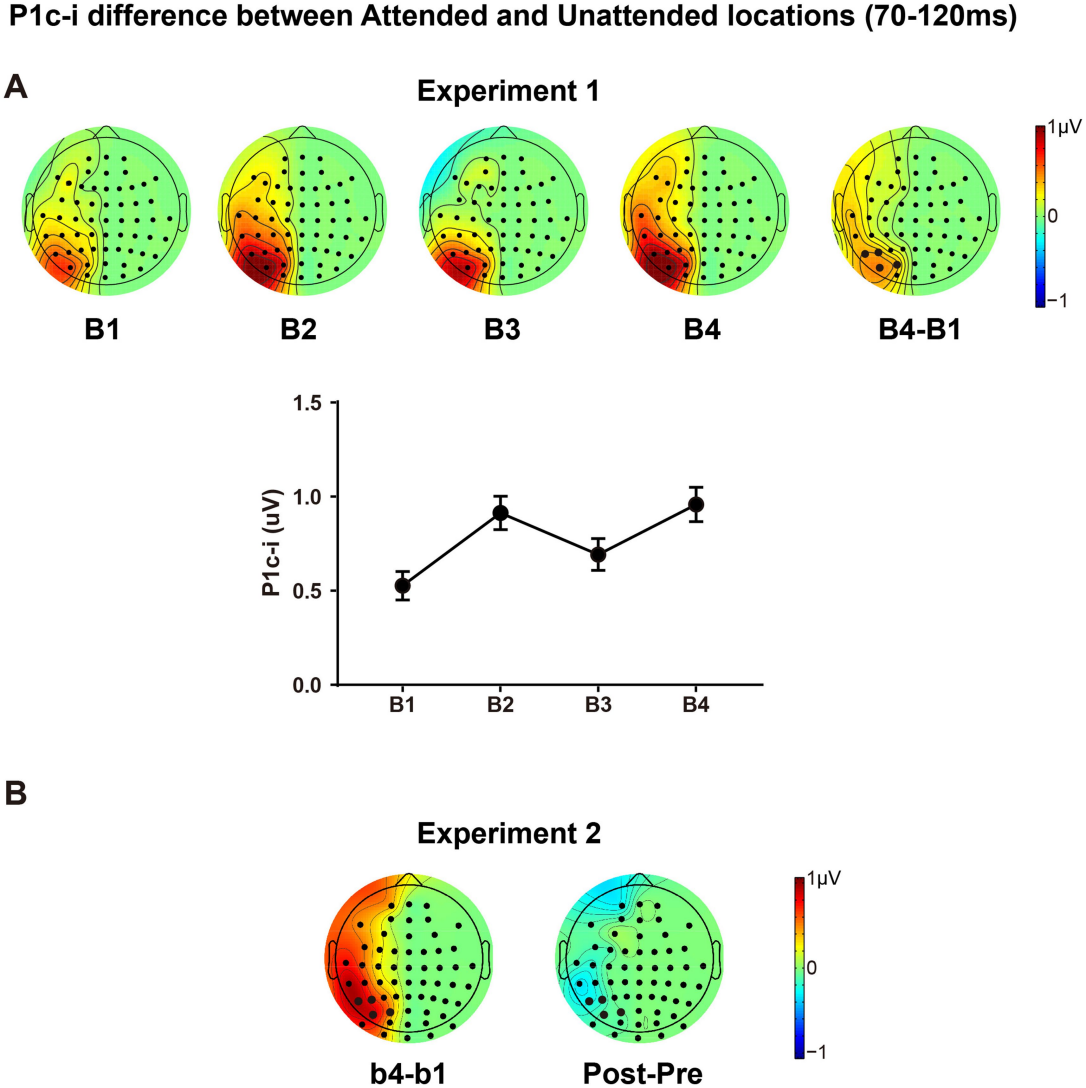
The P1c-i difference between the attended and unattended locations. **(A)** Topographical maps in each training block, the difference map between B4 and B1 and amplitudes of the P1c-i difference along training blocks were presented; the P1c-i was measured at occipital sites highlighted in bold in the time window of 70–120 ms (described in the Data Analysis section); error bars indicate within-subject standard errors. **(B)** The difference map between b4 and b1, and between Posttest and Pretest in Experiment 2.

The present design also allowed us to investigate whether and how spatial attentional effect on early ERPs is modulated by fast learning. As shown in Figure 7, there is a clear attentional effect in P1c-i (70–120 ms) for each training block, as reflected by topographical voltage maps of the P1c-i difference between the attended and unattended locations. This spatial attention effect on P1c-i was enhanced significantly through tens of minutes of training in both Experiment 1 [B1 vs. B4 increment, 0.432 ± 0.131 μV, *t*(15) = 3.290, *p* = 0.005, *d* = 0.823] and Experiment 2 [b1 vs. b4 increment, 0.769 ± 0.250 μV, *t*(15) = 3.081, *p* = 0.008, *d* = 0.770]. However, this fast learning effect on visuospatial attention disappeared when the attended and unattended locations switched in the tests [Pre vs. Post, −0.136 ± 0.185 μV, *t*(15) = −0.734, *p* = 0.474, *d* = −0.184], which is consistent with the location specificity of fast PL.

## 4 General discussion

Combining perceptual learning and sustained spatial attention paradigms, the present study investigated the cognitive neural mechanisms underlying location specificity of fast PL in a Vernier task. We found that tens of minutes of training induced changes of ERP activities at early stages of visual cortical processing. The modifications include not only a facilitation effect specific to the trained location (i.e., the target/attended location during the training), but also a suppression effect specific to the untrained location (i.e., the distractor/unattended location during the training). These two learning-induced effects, as reflected by the increase of late P1c-i (~ 95–120 ms) and the decrease of early P1c-i (~ 75–95 ms) respectively, involve different cognitive neural mechanisms. The late P1c-i enhancement appeared when the trained location was attended during the training but disappeared when it was no longer attended in the test, indicating that this fast PL-induced facilitation may reflect changes in voluntary allocation of attention to the trained location. By contrast, the early P1c-i suppression was observed regardless of whether the untrained location was currently attended or not, indicating that it may represent fast PL-induced changes of automatic/involuntary visual processing at the untrained location. To our knowledge, the present study is the first to reveal both the fast learning-associated changes of brain activities specific to the target location and those specific to the distractor location.

Previous studies have shown that PL has a close relationship with attention (e.g., Ahissar and Hochstein, 1993; Donovan and Carrasco, 2018; Qu et al., 2014, 2017; Szpiro and Carrasco, 2015; Zheng et al., 2007; see Ahissar and Hochstein, 2004; Dosher and Lu, 2017; Watanabe and Sasaki, 2015 for reviews). In most studies showing PL effects on early ERPs (e.g., P1: Wang et al., 2010; Xi et al., 2020; C1: Ahmadi et al., 2018; Pourtois et al., 2008; but see Qu et al., 2014) or low-level visual cortex (e.g., Furmanski et al., 2004; Jia et al., 2020; Schwartz et al., 2002; Yotsumoto et al., 2008), it is unknown whether the effects reflect modulations in voluntary attentional deployment or involuntary visual processing. The present study focused on the early ERP changes induced by fast PL, and further dissociated two location-specific effects involving different cognitive neural mechanisms. Different from the conventional method of analyzing the learning-related changes in original ERPs, the present study adopted a new approach which measured the location-specific PL effects on early contra-minus-ipsilateral ERP waves. As stated in the Method section and the Supplementary file, this new approach could eliminate the overlapping confounds from high-level cortical processing and/or location-nonspecific learning effects to a great extent, which allows us to reveal reliable location-specific PL effects on early ERPs originating from early visual cortex at the trained and untrained locations, respectively. It advances our understanding beyond previous fMRI (e.g., Furmanski et al., 2004; Jia et al., 2020; Yotsumoto et al., 2008) and ERP studies (e.g., Pourtois et al., 2008; Qu et al., 2014; Xi et al., 2020; Zhang et al., 2013) on the underlying mechanisms of location-specific PL, since most measured training-induced changes at the trained relative to the untrained location (i.e., the untrained condition as a baseline). Through this novel method, we obtained consistent and clear results (as reflected in P1c-i effects) in two experiments.

It is interesting that the P1c-i involves two distinct processes (in separate time windows) associated with fast PL. The finding that the P1c-i contained early and late subcomponents (replicated in two experiments) is consistent with some previous studies. For example, source analyses in the human ERP study (Di Russo et al., 2003) showed that the earliest spatial attentional effects on the P1 wave during 80–130 ms could be mainly accounted by two contralateral dipoles; while the source waveform of the early contralateral P1 dipole (located in dorsal extrastriate visual cortex) started at ~80 ms, that of the late contralateral P1 dipole (located in ventral extrastriate visual cortex) began at 100–110 ms. We speculate that the present early and late P1c-i effects might reflect modulations of neural activities originating from different visual cortical areas and involve different types of synapses with distinct plasticity characteristics underlying different cognitive neural processes (i.e., the early part being automatic/involuntary and the late part being voluntary). Considering that the P1 during 75–120 ms was mainly distributed at contralateral sites, we propose that the learning effect observed in the P1c-i mainly reflect changes in the contralateral P1 originating from contralateral extrastriate visual cortex. One might argue that the early P1c-i also involved changes of the first ERP component C1 originating from V1. However, since the C1 usually distributes maximally at or near the midline occipital sites (e.g., slightly ipsilateral to the midline for upper visual field stimuli, Di Russo et al., 2003; the present Figure 3A shows the C1 at ipsilateral sites but not at contralateral sites, which might be largely due to the overlapping of contralateral P1), we speculate that the C1 effect (if there is) would be largely concealed by the present contra-minus-ipsilateral ERP analyses. Nevertheless, we could not rule out a possible C1 effect in the early P1c-i time window. That is, the decrease of early P1c-i might involve both decrease of contralateral P1 and decrease of ipsilateral C1 (note that the ipsilateral C1 has a negative polarity). It is worth noting that, even if a part of the early P1c-i effect was due to a C1 effect, the conclusion that fast PL induces location-specific suppression at early stages of visual cortical processing still holds.

The late P1c-i enhancement appeared when the trained location was attended during the training but disappeared when it was no longer attended in the test, indicating that this fast PL-induced facilitation may reflect changes in voluntary allocation of attention to the trained location. Our previous PL study of a central grating discrimination task (Wang et al., 2010) showed that, tens of minutes of training can enhance the amplitude of P1 (~ 100 ms) evoked by the central target gratings. Here, by using peripheral gratings as the training stimuli, we further reveal that the fast learning-associated P1 enhancement (reflected in the late P1c-i) is specific to the trained target location. We speculate that tens of minutes of active training might modulate the strength of functional connectivity between higher brain centers related to attentional control (e.g., frontal–parietal areas) and early visual cortex processing the trained information (e.g., occipital areas; Hopfinger et al., 2000; Qu et al., 2010); this fast PL induced facilitation of top-down selective attention can be preserved only when the trained location is attended and contributes to the behavioral improvement specific to the trained location. By contrast, the decrease of early P1c-i was observed regardless of whether the untrained location was currently attended or not, indicating that it may represent fast PL-induced changes of automatic/involuntary visual processing at the untrained location. Since the early P1c-i effect appeared at the untrained but not the trained location, it is less likely this effect simply reflected sensory adaptation due to stimulus repetition regardless of whether the stimuli are attended/relevant or not during the training (it would be useful to further clarify this point by presenting the same visual stimuli without a PL task and to examine whether stimulus repetition induces decrease of P1c-i). We speculate that this fast PL-induced involuntary suppression might reflect habituation of the neural process automatically and selectively responding to the distractors during the training. This neural habituation is induced by repetition of *irrelevant* stimuli, which is important for avoiding the interference of distractors presented outside the top-down attentional window and is helpful for allocating attention to the target and relevant information. This neural habituation is developed over tens of minutes and cannot be quickly eliminated (e.g., it was preserved even when the untrained location was currently the target location and required active allocation of attentional resources), leading to poor behavioral performance at the untrained location. Thus, though the two fast PL-induced location-specific ERP effects reflected distinct cognitive neural mechanisms, they may contribute together to the location specificity of PL in behavior.

The present findings may be contrasted with recent studies of statistical learning (SL) which also reported modifications of early visual processing induced by fast learning. Using visual search paradigms, recent studies of SL reported an early Pd (~ 100 ms) specific to the frequent distractor location (i.e., the high-probability distractor location; van Moorselaar et al., 2021; Wang et al., 2019), supporting that fast learning may induce distractor suppression at the early stage of visual cortical processing. However, while the early Pd evoked by distractors *increased* with SL, the present distractor-elicited early P1c-i *decreased* with PL, indicating that they involve distinct mechanisms of distractor suppression. A key difference might be that the designs of the training tasks are very different between these studies. The present PL study adopted sustained spatial attention tasks in which targets and distractors were presented asynchronously, at fixed locations throughout the training, which were explicitly known by the subjects. In contrast, the SL studies used visual search tasks in which the target and the salient singleton distractor were presented simultaneously, at randomized locations throughout the training, and the frequent distractor location was unknown by the subjects. Different experimental designs may involve different cognitive neural processes associated with the learning. Besides, the early Pd effects reported in the visual SL studies may be confounded with inter-trial priming (see van Moorselaar et al., 2021 for a similar discussion) which could be excluded in the present study (See *Inter-trial target/distractor effect* in the Results).

The present findings also provide evidence with regard to whether and how the effects of spatial selective attention evolve along training. Using endogenous cueing or sustained attention paradigms, numerous ERP studies have shown that top-down spatial attention could modify early visual cortical processing. The ERPs evoked by stimuli at target/attended locations and those at distractor/unattended locations differ in amplitude as early as 70–120 ms post-stimulus onset (i.e., the well-known spatial attentional effects on the P1 component; Di Russo et al., 2003; Hillyard and Anllo-Vento, 1998; Luck and Kappenman, 2012; Mangun and Hillyard, 1987). The present study further reveals this early visual selection between target and distractor locations could be significantly enlarged by tens of minutes of training (Figure 7). While the dissociable distractor and target processing has been revealed by the studies of selective attention using spatial cueing paradigms (Luck et al., 1994; Talsma et al., 2007; see Luck and Kappenman, 2012 for a review), as reflected by a decreased P1 (60–120 ms) and an increased N1 (140–200 ms), here we observe an early dissociation (within 120 ms) from the PL perspective, providing further evidence supporting that target facilitation and distractor inhibition are not simply different sides of the same coin but are controlled by distinct cognitive mechanisms.

Although both of the two fast PL-induced early ERP effects (i.e., increase of late P1c-i at the trained location and decrease of early P1c-i at the untrained location) are specific to location, it remains unclear whether they are specific to stimulus orientation or task types as well. Our previous fast PL studies (Ding et al., 2003; Qu et al., 2014; Song et al., 2010) showed that the orientation-specific learning effects of ERPs did not appear until ~200 ms post-stimulus onset. Considering that these fast orientation-specific PL studies all adopted easy task during the training or test, and that the early learning or attentional effect on the P1 component was reported in difficult rather than easy training conditions (Ding et al., 2014; Qu et al., 2014; Wang et al., 2010), further studies are needed to clarify whether the early location-specific ERP effects induced by fast learning could transfer to untrained simple stimulus features, such as orientation. In addition, since only one type of task (i.e., Vernier task) was used in the present study, it remains to be clarified in the future whether the P1c-i learning effects appear when the Vernier task is replaced with other PL tasks (e.g., visual discrimination of color, shape, and so on), and whether these location-specific learning effects could transfer between different visual tasks. Addressing this issue is helpful to understand whether the early location-specific fast learning effects reflect modulations of purely spatial attention (e.g., learning occurs at the level of priority map) or lower sensory processing (e.g., learning occurs at the level of feature map or sensory register; Liesefeld and Müller, 2021; Luck et al., 2021).

Similar to many previous studies of spatial attention (e.g., Clark and Hillyard, 1996; Di Russo et al., 2003; Martinez et al., 1999) and/or perceptual learning (e.g., Xi et al., 2020; Zhang et al., 2013), the present study did not monitor subjects’ precise eye positions by eye tracking during the experiment. Instead, we emphasized the importance of eye fixation to each subject and rejected EEG trials with eye movement artifacts to minimize confounds of eye movement. One might argue that the present P1c-i effects may be due to continuous deviation of the fixation point toward the attended stimulus or the deviation of fixation changes across training blocks. However, the present results showed classic early ERPs evoked by peripheral stimuli (i.e., the P1 was larger at the contralateral occipital sites than ipsilateral sites during 70–120 ms, as reflected by P1c-i for both the attended and unattended stimuli) and typical spatial attentional effects on the early ERPs (i.e., the P1 or P1c-i was larger for the attended than the unattended stimuli, Figure 7), both supporting that subjects well fixated on the central fixation point during the experiments. Moreover, if subjects deviated their fixation points to the attended stimuli and/or the deviation changed across training blocks, then (1) the retinal position of the peripheral attended stimuli would vary along training, and the early P1c-i evoked by the peripheral attended stimuli would differ across training blocks; (2) the retinal position of the peripheral stimuli at the untrained location would be different between the training and test blocks, which would induce different effects on the early P1c-i across training blocks and across pre−/post- tests. However, we did not find the early P1c-i training effect at the trained/attended location for both experiments; and the early P1c-i exhibited similar learning effects for the untrained location when it was unattended during the training and was attended during the tests. If the early P1c-i learning effects observed in both Exp 1 and 2 was just a confound of fixation deviation, it would be hard to explain why the deviation of fixation point only affected the early P1c-i of untrained location but not that of the trained location. Taken together, the present P1c-i effects consistently support a learning and/or spatial attention mechanism (as we have discussed earlier) rather than the eye movement or fixation deviation confound. Nevertheless, further studies with eye tracking are needed to completely rule out this confounding factor.

It should be noted that, the present contra-minus-ipsilateral ERP method can eliminate the confounding activities distributed centrally or bilaterally (e.g., CNV or response related activities from previous trials, and the midline P1 evoked by current stimuli), but not the high-level brain activities with scalp distributions lateral to the stimulus location. Studies have shown the existence of retinotopic maps in temporal, parietal, and even frontal cortex (Ramezanpour and Fallah, 2022); and these high-level cortical areas could be quickly activated by salient stimuli (Lamme and Roelfsema, 2000), possibly through subcortical pathway and important for automatic attention orienting (Mulckhuyse and Theeuwes, 2010; Veale et al., 2017). Note that in both experiments of the present study, contra-minus-ipsilateral ERPs evoked by stimuli at the untrained location showed significant changes during 75–95 ms not only at the occipital sites (i.e., the early P1c-i effect) but also at anterior scalp sites (e.g., CP5/6; Topographic maps in Figures 3D, 6B,D). These early anterior effects might originate from the high-level retinotopic cortical areas and reflect training-associated decrease of involuntary attention to the untrained location. A speculation is that anticipation of stimulus location may underlie the early ERP learning effects, since the stimuli were presented at the trained or untrained location in a pseudo-random order in the present study (i.e., the current stimulus was more likely to appear at the location different from the preceding stimulus). However, we did not find reliable learning-induced anticipatory effect on the contra-minus-ipsilateral ERPs for either the trained or the untrained location. Further studies are needed to clarify whether the P1c-i effects are related to anticipatory spatial attention mechanism.

So far, location specificity of PL has been observed not only in tens of minutes of training (fast learning, e.g., Fahle et al., 1995; Shiu and Pashler, 1992) but also in extensive training over several days (slow learning, e.g., Harris et al., 2012; Karni and Sagi, 1991; Yotsumoto et al., 2008). The present study focused on location specificity of fast PL. It remains unclear whether similar mechanisms underlying that of slow PL. It has been proposed that fast learning and slow learning involves different mechanisms (Karni and Sagi, 1993); while fast learning is important for establishing and maintaining neural processing routines for the perceptual task, slow learning involves both increased stimulus representation in lower sensory cortex and decreased attentional modulation in higher level brain areas (Qu et al., 2010). Based on present findings and literatures, we further propose that the neural processing routines for the perceptual task established by fast PL involve not only task rules or familiarity, but also selective attention to the trained target and/or its location, orientation et al. (Ding et al., 2023; Qu et al., 2014; Su et al., 2014). Through enhancing low-level visual perception across many dimensions (Carrasco, 2011; Carrasco and Barbot, 2015; Hillyard and Anllo-Vento, 1998; Luck and Kappenman, 2012), such selective attention (endogenous or exogenous; spatial, feature and/or object attention) may facilitate PL process (e.g., Zheng et al., 2007; Donovan and Carrasco, 2018; Donovan et al., 2020; Hung and Carrasco, 2021; Szpiro and Carrasco, 2015). In some conditions, the selective attentional processing, together with sensory adaptation induced by stimulus repetition (e.g., Harris et al., 2012), may be essential for the slow formation of visual cortical representation for (and involuntary attention capture by) the trained stimuli (Qu et al., 2017; Hu et al., 2019). Future studies may apply the present approach to investigate the location-specific mechanism of slow PL (e.g., to test whether slow learning developed over days can enhance involuntary attention and/or sensory processing to the trained location or stimuli, which may also account for location specificity of PL).

Similar to many prior PL studies (e.g., Gutnisky et al., 2009; Karni and Sagi, 1991; Mastropasqua et al., 2015; Wang et al., 2012), the present study revealed location specificity of PL when there were highly-visible distractors presented at the untrained location during the training. Since only strong (suprathreshold) stimuli could be effectively inhibited by the brain whereas weak stimuli could not (Tsushima et al., 2006; Hu et al., 2019), suppression of highly-visible distractors might account for learning-induced suppression at the untrained location in the present study. This is in accordance to a prior study (Xiong et al., 2016) which showed no specificity of learning when the distractors presented at the untrained location during the training were invisible. However, many other studies showed the location specificity of PL when there were no distractors presented at the untrained location during training (e.g., Crist et al., 1997; Fahle et al., 1995; Mastropasqua et al., 2015). Specifically, in the study of Mastropasqua et al. (2015), behavioral performance showed similar learning specificity in conditions with or without distractors presented at the untrained location. Thus, it remains an interesting and open question whether the location specificity of PL involves suppression to an untrained location in the paradigms without stimulus presentation at an untrained location during training.

As we have mentioned before, we are not sure whether the early P1c-i effect involves ipsilateral C1 reduction as well. Actually, it is a long-debated and unsolved question whether the initial feedforward processing in V1 (as reflected by the C1 component) could be modulated by top-down factors such as spatial attention (see Baumgartner et al., 2018; Ding, 2018; Qu and Ding, 2018; Slotnick, 2018 for reviews and comments in the special issue “Attentional modulation of early visual areas” edited by Slotnick, 2018) or perceptual learning (e.g., Ahmadi et al., 2018; Bao et al., 2010; Pourtois et al., 2008; Zhang et al., 2015; Qu et al., 2014; Wang et al., 2016). One difficulty is how to dissociate the C1 and overlapping confounds (e.g., slow waves from preceding trials and/or P1 components). More sophisticated designs and innovative approaches are needed in future studies to address this difficult but important question.

## 5 Conclusion

The present study shows that fast perceptual learning induces both location-specific facilitation and location-specific suppression at the early stages of visual cortical processing. While the facilitation effect indicates a more efficient allocation of voluntary attention to the trained location, the suppression effect may reflect learning-associated involuntary suppression of visual processing at the untrained location. The facilitation of trained location and suppression of untrained location may involve distinct cortical plasticity mechanisms, contributing jointly to the location specificity of perceptual learning in behavior. Although there are still many intriguing issues that require further research to clarify, the present study provides a valuable approach in the study of location-specificity of PL and brings new insights into the relationship between perceptual learning and spatial attention.

## Data Availability

The raw data supporting the conclusions of this article will be made available by the authors, without undue reservation.
