# Supplementary material for: Fast perceptual learning induces location-specific facilitation and suppression at early stages of visual cortical processing
Source: Front Hum Neurosci. 2025 Jan 17;18:1473644. doi: 10.3389/fnhum.2024.1473644 (PMC11782211; doi:10.3389/fnhum.2024.1473644)
Supplement: Supplementary file 1 [file Data_Sheet_1.docx]

Supplemental File

In the supplemental file, we elaborated on why the conventional approach (analyses of the original ERPs) is not suitable for the present study.

- First, the original ERPs in the present study contained several overlapping confounds from high-level cortical processing, some of which are common in fast PL studies (as well as in some early visual attention studies). The confounds include slow-wave activities from preceding trials and training-induced location-nonspecific ERP effects, both of which are not the interests of the present study. These overlapping confounds (details below) might even change along training, making it almost impossible to get reliable or interpretable location-specific PL effects on the original ERPs. Fortunately, the scalp distributions of these confounds were generally unrelated to the stimulus locations, thus analyses on the contra-minus-ipsilateral ERPs could eliminate the confounds to a great extent and get clearer and more meaningful location-specific PL effects on early ERPs.

Two major confounds overlapped in the early original ERPs:

(1) Slow-wave activities from preceding trials

The slow-wave activities include response-related ERPs and anticipatory waveforms like the contingent negative variation. As shown in **Supplemental Figure** **1A** and **1B**, the slow-wave activities overlapped with the early ERPs at both ipsilateral and contralateral sites, and seemed to change with training as well. Thus, it’s almost impossible to exclude the contamination of these confounds when examining how the P1 was modulated by PL through the original waves.

Fortunately, these slow-wave activities from preceding trials ought to be insensitive to the location of eliciting stimuli, with bilateral or central scalp distributions. First, for any given target location (attend-repeat or attend-opposite), the response would be made by left or right index finger with equal probabilities. Since the left and right responses were well matched, the activities related to response should exhibit a bilateral distribution to any target location. Second, previous studies showed that the anticipatory waveforms (e.g., CNV) were centrally distributed, with a maximum at/near the midline (see Luck & Kappenman, 2012, pp. 244-245; Mangun, 2013, pp.45-57). Given that these overlapping slow-wave activities are location insensitive, they should make little contribution to the contra-minus-ipsilateral ERPs (as shown in **Supplemental Figure** **1C** and **1D**, as well as **Figure 3** and **5 in the manuscript**).


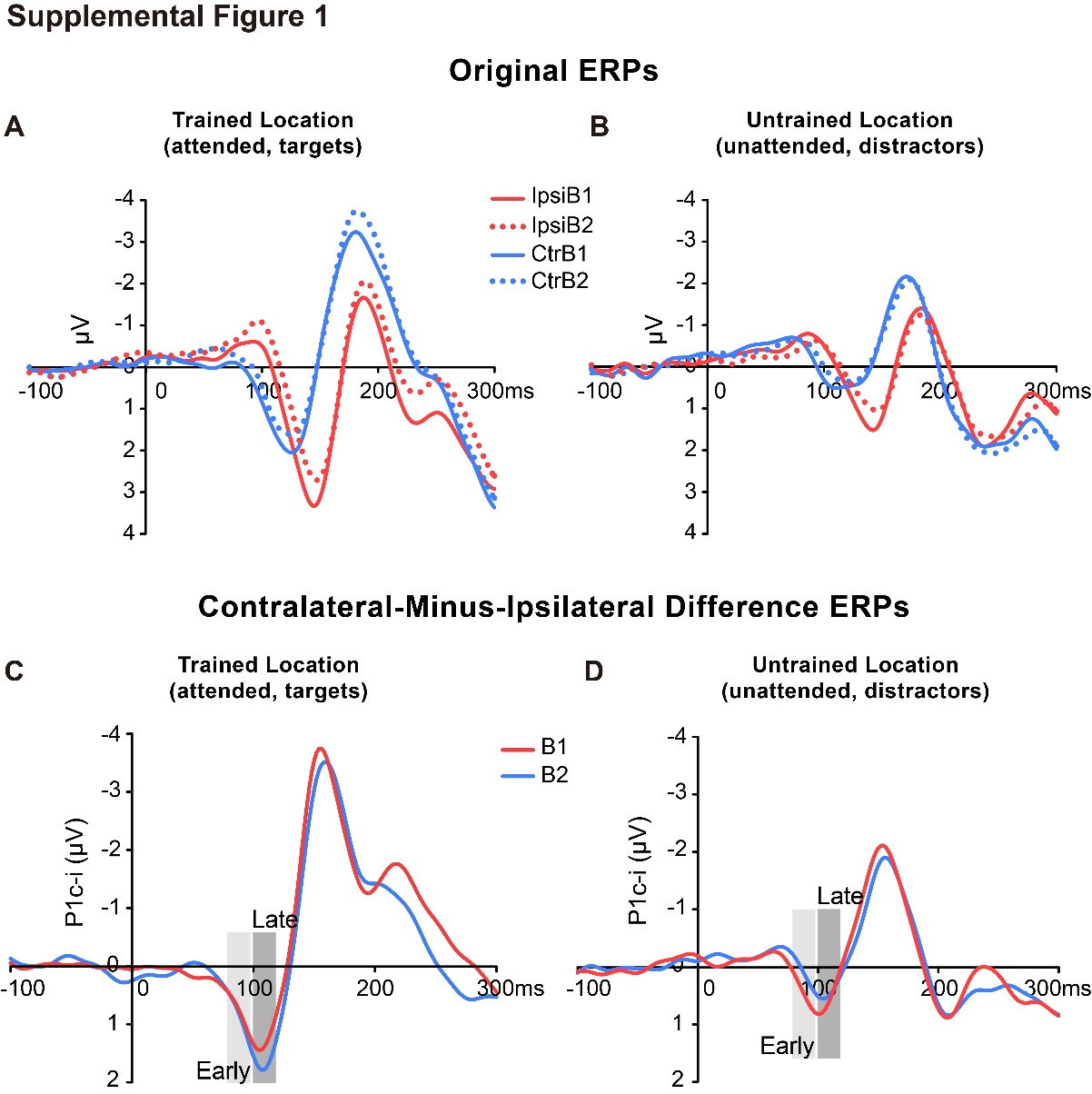


**Supplementary Figure 1** Grand average ERPs at lateral occipital sites in Experiment 1. **(A, B)** Original ERP waveforms elicited by Vernier stimuli as targets at the trained location **(A)** or as distractors at the untrained location **(B)** at electrodes contralateral (blue lines) and ipsilateral (red lines) to the stimulus locations in the training Block1 (solid lines) and Block2 (dashed lines). Waveforms contralateral to the stimulus locations were collapsed across PO8, PO4, P8, and P6, and the ipsilateral waves were collapsed across PO7, PO3, P7, and P5. As shown in **(A)** and **(B)**, slow-wave activities from preceding trials overlapped with early ERPs at both ipsilateral and contralateral sites, and the overlapping also changed with training, so it’s hard to isolate those training-induced changes on the original early ERPs. **(C, D)** Contralateral-minus-ipsilateral difference waveform elicited by targets at the trained location **(C)** or distractors at the untrained location **(D)** in the training Block1 (red lines) and Block2 (blue lines). Shaded rectangles indicate two time intervals (i.e., 75-95 ms and 100-120 ms) that were used to measure location-specific P1c-i effects. As shown in **(C)** and **(D)**, contra-minus-ipsilateral ERPs could eliminate those overlapping slow-wave activities to a great extent and get reliable perceptual learning effects.

(2) location-nonspecific training effect: midline P1 (P1m) effect

As shown in **Supplemental Figure** **2A** and **2B**, for both the trained and untrained locations, training induced significant negative deflections in the original ERPs during 80-150 ms, with a broad distribution over central and posterior scalp sites. Since this training effect showed maximum amplitudes at midline scalp sites (CPz and Pz), we call it ‘midline P1’ or ‘P1m’ effect (the “P1m” was also reported and discussed in some studies about exogenous attention, see Fu et al., 2009; Dassanayake et al., 2016; and Baumgartner et al., 2018 for a comment on these studies; it remains unclear whether the present P1m and the P1m reported earlier shared some common mechanisms, although they showed similar spatial distributions and time windows).

Like the slow-wave activities above, the P1m effect shown in the original ERPs was midline-symmetric distributed, not specific to the trained or untrained location. The P1m is a strong effect associated with training, and has been observed in several fast-learning studies in our lab (not published yet). Although we are not sure what this training effect actually reflects, it’s clear that it was unrelated to location-specific PL, and thus not the interest of the present study.

Considering that the time course and scalp distribution of the P1m effect overlapped with the location-specific P1c-i effects (especially the late part) to a great extent, if we examine the learning effects on the original ERPs, the location-specific PL effects would be contaminated and even concealed by the location-nonspecific P1m effect. Instead, analyses on contra-minus-ipsilateral ERPs could well eliminate the confound of P1m effect, leaving location-specific P1c-i effects retained.


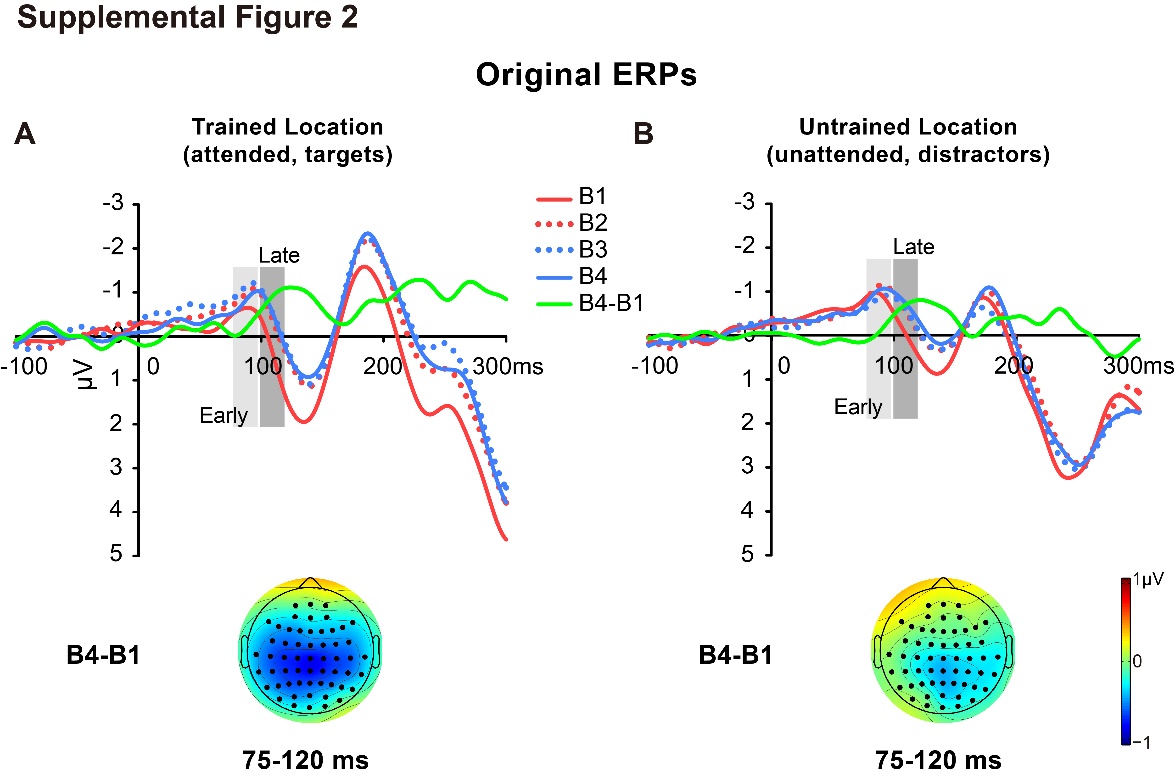


**Supplementary Figure 2** Grand average ERPs at midline occipital sites and voltage topographies in Experiment 1. **(A, B)** Original and difference ERPs elicited by targets at the trained location **(A)** and by distractors at the untrained location **(B)** during the training. Original ERPs and ERP difference waves between Block1 and Block4 were collapsed across midline electrodes (Pz and POz). Shaded rectangles indicate two time intervals (i.e., 75-95 ms and 100-120 ms) that were used to measure location-specific P1c-i effects. Topographies of the time window (75-120 ms) were plotted based on ERP difference waves constructed by subtracting B1 from B4 to show the midline P1 effect. Topographies of ipsilateral and contralateral activities were projected to the left and right side of the head, respectively. As shown in **(A)** and **(B)**, the midline P1 is a strong effect associated with training, and appears in both the trained and untrained locations. Considering that the time course and scalp distribution of the midline P1 effect overlapped with the location-specific P1c-i effects (75-120 ms), contra-minus-ipsilateral ERPs were analyzed in the present study to minimize the overlapping of the midline P1 effect.

Besides the two major confounds above, the contra-minus-ipsilateral approach could also well eliminate the alpha residues from spontaneous EEG, a common confound especially under task conditions where the trial number is relatively limited.

- Second, one might recommend another approach to reveal the learning effect, in which the original ERPs to a specific stimulus is compared across attended and unattended conditions. However, this method still has some limitations in reducing signal overlap in the present study.

In the present study, to avoid repeated appearance of stimuli at the same location for more than 3 consecutive trials, the stimuli were presented at the trained or untrained location in a pseudo-random order. This led to the proportion of stimuli at the trained vs. untrained location in preceding trials being different between the current trained/attended and untrained/unattended trial conditions. As a result, the overlapping (slow-wave activities) from preceding trials might be also different between the two conditions, since the late slow-wave activities evoked by attended and unattended stimuli were not the same. Specifically, while the overlapping of slow-wave activities on the early original ERPs at the trained location becomes more negative at both the contra- and ipsi-lateral sites across training (**Supplemental Figure** **1A**), that at the untrained location becomes more positive across training (**Supplemental Figure** **1B**). Due to such a confound, a conventional approach to directly compare the original ERPs to a specific stimulus between these two locations is not suitable in reducing overlapping and revealing the change on early visual ERPs (e.g., C1 and lateral P1).

Even if overlapping is identical across the trained and untrained locations, which would make a conventional analysis (i.e., directly comparing the original ERPs between these two locations) effective in reducing confounds, such analysis would still fail to track ERP changes during training at the trained and untrained locations respectively. The present study contributes to a deeper understanding of cognitive neural mechanisms underlying location-specific PL at both trained and untrained locations, particularly highlighting a learned suppression of visual processing at the untrained location. Since most previous studies (e.g., fMRI studies, Furmanski et al., 2004; Yotsumoto et al., 2008; Jia et al., 2020; ERP studies, Pourtois et al., 2008; Zhang et al., 2013; Xi et al., 2020) measured training-induced changes at the trained relative to the untrained location (i.e., the untrained condition as a baseline), it is not clear whether location specificity involves both facilitation of trained locations and suppression of untrained locations. Instead, the new method (i.e., analysis on contra-minus-ipsilateral ERP waves) reduced signal overlapping, which helps delineate fast learning-associated changes in brain activities, even specific to the untrained location.

Taken together, we believe that analyses on contra-minus-ipsilateral ERPs rather than the original ERPs might be a more suitable method for the aim of the present study (i.e., revealing location-specific PL effect in early ERPs over lower visual cortical areas). Importantly, through this novel method, we obtained consistent results (i.e., early P1c-i reduction at untrained location and late P1c-i increase at trained location) in two experiments, supporting this new method is indeed a meaningful/reliable approach in ERP studies to reveal fast PL associated changes of activities in lower visual cortical areas.

**References in the Supplemental File**

Baumgartner, H. M., Graulty, C. J., Hillyard, S. A., & Pitts, M. A. (2018). Does spatial attention modulate the C1 component? The jury continues to deliberate. *Cognitive neuroscience*, *9*(1-2), 34-37.

Dassanayake, T. L., Michie, P. T., & Fulham, R. (2016). Effect of temporal predictability on exogenous attentional modulation of feedforward processing in the striate cortex. *International Journal of Psychophysiology*, *105*, 9-16.

Furmanski, C. S., Schluppeck, D., & Engel, S. A. (2004). Learning strengthens the response of primary visual cortex to simple patterns. *Current Biology*, *14*(7), 573-578.

Fu, S., Huang, Y., Luo, Y., Wang, Y., Fedota, J., Greenwood, P. M., & Parasuraman, R. (2009). Perceptual load interacts with involuntary attention at early processing stages: event-related potential studies. *NeuroImage*, *48*(1), 191–199.

Luck, S. J., & Kappenman, E. S. (2012). Negative slow waves as indices of anticipation: the Bereitschaftspotential, the contingent negative variation, and the stimulus-preceding negativity. In Brunia, C. H., van Boxtel, G. J., & Böcker, K. B. (Eds.), *The Oxford handbook of event-related potential components* (pp. 244-245). Oxford university press.

Jia, K., Zamboni, E., Kemper, V., Rua, C., Goncalves, N. R., Ng, A. K. T., ... & Kourtzi, Z. (2020). Recurrent processing drives perceptual plasticity. *Current Biology*, *30*(21), 4177-4187.

Mangun, G. R. (2013). Sequential Effects in the Central Cue Posner Paradigm–On-line Bayesian Learning. In Arjona, A., & Gómez, C. M. (Eds.), *Cognitive electrophysiology of attention: signals of the mind* (pp. 45-57). Academic Press.

Pourtois, G., Rauss, K. S., Vuilleumier, P., & Schwartz, S. (2008). Effects of perceptual learning on primary visual cortex activity in humans. *Vision research*, *48*(1), 55-62.

Yotsumoto, Y., Watanabe, T., & Sasaki, Y. (2008). Different dynamics of performance and brain activation in the time course of perceptual learning. *Neuron*, *57*(6), 827-833.

Xi, J., Zhang, P., Jia, W. L., Chen, N., Yang, J., Wang, G. T., ... & Huang, C. B. (2020). Multi-stage cortical plasticity induced by visual contrast learning. *Frontiers in Neuroscience*, *14*, 1-15.

Zhang, G., Cong, L., Song, Y., & Yu, C. (2013). ERP P1-N1 changes associated with Vernier perceptual learning and its location specificity and transfer. *Journal of vision*, *13*(4), 1-13.
